# Targeting cancer stemness mediated by BMI1 and MCL1 for non‐small cell lung cancer treatment

**DOI:** 10.1111/jcmm.17453

**Published:** 2022-07-06

**Authors:** Erh‐Hsuan Lin, Jhen‐Wei Hsu, Ting‐Fang Lee, Chiung‐Fang Hsu, Tsung‐Hsien Lin, Yi‐Hua Jan, Hsiang‐Yi Chang, Chun‐Ming Cheng, Hui‐Jan Hsu, Wei‐Wei Chen, Bo‐Hung Chen, Hsing‐Fang Tsai, Jung‐Jung Li, Chi‐Ying Huang, Shih‐Hsien Chuang, Jia‐Ming Chang, Michael Hsiao, Cheng‐Wen Wu

**Affiliations:** ^1^ Institute of Biomedical Sciences Academia Sinica Taipei Taiwan; ^2^ Institute of Microbiology and Immunology National Yang Ming Chiao Tung University Taipei Taiwan; ^3^ Institute of Clinical Medicine National Yang Ming Chiao Tung University Taipei Taiwan; ^4^ Institute of Biochemistry and Molecular Biology National Yang Ming Chiao Tung University Taipei Taiwan; ^5^ Genomics Research Center Academia Sinica Taipei Taiwan; ^6^ Department of Pharmacology, Development Center for Biotechnology Institute for Drug Evaluation Platform Taipei Taiwan; ^7^ Department of Medicinal Chemistry, Development Center for Biotechnology Institute of Pharmaceutics Taipei Taiwan; ^8^ Institute of Biopharmaceutical Sciences National Yang Ming Chiao Tung University Taipei Taiwan

**Keywords:** BMI1, cancer stemness, MCL1, NSCLC, small‐molecule therapy

## Abstract

Lung cancer is the leading cause of cancer‐associated death, with a global 5‐year survival rate <20%. Early metastasis and recurrence remain major challenges for lung cancer treatment. The stemness property of cancer cells has been suggested to play a key role in cancer plasticity, metastasis and drug‐resistance, and is a potential target for drug development. In this study, we found that in non‐small cell lung cancer (NSCLC), BMI1 and MCL1 play crucial roles of cancer stemness including invasion, chemo‐resistance and tumour initiation. JNK signalling serves as a link between oncogenic pathway or genotoxicity to cancer stemness. The activation of JNK, either by mutant EGFR or chemotherapy agent, stabilized BMI1 and MCL1 proteins through suppressing the expression of E3‐ubiquitin ligase HUWE1. In lung cancer patient samples, high level of BMI1 is correlated with poor survival, and the expression of BMI1 is positively correlated with MCL1. A novel small‐molecule, BI‐44, was developed, which effectively suppressed BMI1/MCL1 expressions and inhibited tumour formation and progression in preclinical models. Targeting cancer stemness mediated by BMI1/MCL1 with BI‐44 provides the basis for a new therapeutic approach in NSCLC treatment.

## 1 BACKGROUND

Lung cancer is the leading cause of cancer‐associated death, with a global 5‐year survival rate <20%.^1^ Non‐small cell lung cancer (NSCLC) accounts for ~75% of lung cancers, among which lung adenocarcinoma (LAC) is the most common (~40%) histological subtype. LAC is frequently associated with activating epidermal growth factor receptor (EGFR) mutations, and chemotherapy has limited efficacy.^2^ Although EGFR Tyrosine Kinase Inhibitors (EGFR‐TKIs) has offered an improved progression‐free survival in lung cancer patients with EGFR mutations, drug resistance invariably occurs.^3^ Cancer stem cell (CSC) has been suggested to play a key role in cancer plasticity, metastasis and drug‐resistance.^4^, ^5^ Although the origin and exact definition of CSCs may be still controversial, it is generally accepted that stemness pathways are activated in these cells, which interact with oncogenic pathways and drive tumour initiation and drug resistance.^6^ Development of agents targeting cancer stemness has been considered a promising strategy for cancer treatment.^4^, ^5^

B‐cell‐specific Moloney murine leukaemia virus integration site 1 (BMI1), a member of the Polycomb Repressive Complex 1 (PRC1), is required for the self‐renewal in a variety of adult stem cells including the lung.^7^, ^8^ BMI1 gene amplification or protein overexpression has also been found in various cancer types.^9^ BMI1 expression has been linked to the promotion of stemness properties of tumour cells, including to tumour initiation, cell proliferation, epithelial–mesenchymal transition (EMT), invasion, repression of apoptosis or senescence and drug resistance.^8^, ^9^, ^10^, ^11^ In lung cancer, however, the role of BMI1 has not been fully characterized. Although clinical studies confirmed the association of BMI1 with a poor survival in lung cancer patients,^12^, ^13^ how BMI1 drives the tumorigenesis in lung cancer remains elusive. While BMI1 is a potential therapeutic target, the development of BMI1 inhibitor for cancer treatment is still in the beginning stage. The first BMI1 inhibitor PTC‐209 was reported in 2014,^14^ which showed promising anti‐cancer effect in pre‐clinical model of several types of tumours.^14^, ^15^, ^16^ Unfortunately, PTC‐209 has not entered clinical trials because of poor pharmacokinetic properties.^17^ Another BMI1 inhibitor, PTC‐596, which demonstrated in vivo anti‐leukaemia activity and showed a favourable safety profile,^17^, ^18^ has recently entered Phase 1 clinical trials.

Myeloid cell leukaemia 1 (MCL1) is a pro‐survival member of the B‐cell lymphoma 2 (BCL‐2) family of proteins, frequently overexpressed or genetically amplified in several types of tumours.^19^, ^20^, ^21^, ^22^, ^23^ MCL1 blocks the progression of apoptosis by binding and sequestering BAK, BAX and/or other pro‐apoptotic members. Overexpression of MCL1 has been suggested as a major cause of resistance to radio‐ and chemo‐therapies.^20^, ^21^ Besides the anti‐apoptotic function, MCL1 also has a pivotal role in the maintenance of survival and self‐renewal in both malignant lymphocytes and haematopoietic stem cells.^19^, ^24^ MCL1 is a critical and specific regulator in the homeostasis of early haematopoietic progenitors. Knockdown of MCL1 specifically reduced the in vivo self‐renewal function of human haematopoietic stem cells.^24^, ^25^, ^26^ Meanwhile, MCL1 overexpression can promote malignant transformation of haematopoietic stem and progenitor cells.^27^ MCL1 has a very short half‐life and is tightly regulated by multiple pathways in transcriptional, translational or post‐translational levels.^19^, ^21^ At least six E3‐ubiquitin ligases have been identified to regulate MCL1 stability.^19^ Although MCL1 has a prominent cytosolic localization, where it regulates the mitochondrial pathway of apoptosis, nuclear localization of MCL1 has also been detected and suggested to be involved in cell cycle regulation and DNA damage response. Loss of MCL1 leads to genomic instability and impairs DNA double‐strand break repair.^28^, ^29^, ^30^, ^31^

C‐Jun N‐terminal kinase (JNK) is a master protein kinase that regulates many physiological processes, including stress and inflammatory responses, morphogenesis, cell proliferation, differentiation, survival and death.^32^ The JNK kinase family includes three proteins (JNK1, JNK2 and JNK‐3) encoded by three separate genes.^33^ JNK1 and JNK2 are expressed ubiquitously, while JNK3 is predominantly in the brain, testis and heart. JNK signalling pathway can be activated in response to extracellular stimuli especially drug treatment, such as doxorubicin or cisplatin‐induced DNA damage.^34^, ^35^ Because of the complexity of downstream signalling involved, the activation of JNK can be a double‐edged sword which can either potentiate or inhibit oncogenesis, depending on the cellular context.^35^, ^36^, ^37^ In lung cancer, the role of JNK was relatively less studied. Previous researches suggested that JNK signalling play a pro‐oncogenic role in lung cancer.^38^, ^39^, ^40^ However, the exact mechanism and function of JNK signalling remain elusive.

In the current study, we showed that in non‐small cell lung cancer (NSCLC), JNK signalling is a link between oncogenic pathway or environment stress to cancer stemness. The activation of JNK, either by EGFR or chemotherapy agent, stabilized and enhanced BMI1 and MCL1 protein expressions, which promoted self‐renewal and chemo‐resistance in LAC cells. A novel small‐molecule BI‐44 was synthesized, which suppressed BMI1/MCL1 expression and showed significant anti‐tumour effect in preclinical models, providing a new and promising approach for NSCLC treatment.

## 2 METHODS

### 2.1 Cell culture

Human LAC cell lines A549 (ATCC CCL‐185), H1975 (ATCC CRL‐5908), HCC827 (ATCC CRL‐2868), H3255 (ATCC CRL‐2882) and human non‐tumour lung epithelial cell line BEAS‐2B (ATCC CRL‐9609) were purchased form American Type Culture Collection (ATCC, VA, USA). PC9 and CL1‐5 were kindly provided by Dr. Pan‐Chyr Yang (National Taiwan University).

### 2.2 Animal experiment

Five‐week‐old nude mice (BALB/cAnN.Cg‐Foxn1nu/CrlNarl) were purchased from National Laboratory Animal Center (Taipei, Taiwan), and maintained in the animal facility of Institute of Biomedical Sciences (IBMS) of Academia Sinica (Taipei, Taiwan). Five‐week‐old SCID mice (CB17.Cg‐Prkdc^scid^Lyst^bg‐J^/CrlBltw) were purchased from BioLasco Inc. (Taipei, Taiwan), and maintained in the animal facility of Development Center of Biotechnology (DCB, Taipei, Taiwan). All animal experiment protocols are reviewed and approved by Institutional Animal Care and Utilization Committee (IACUC) in Academia Sinica (14–02‐647), DCB (2015‐R501‐015‐a, −g) and National Taiwan University (NTU105‐EL‐00137).

### 2.3 Clinical samples

Clinical NSCLC samples were collected with IRB approval (KMUHIRB‐E[I]‐20,160,099) from the Kaohsiung Medical University Hospital and fixed in formalin and embedded in paraffin before archiving. Archived specimens, with follow‐up time up to 200 months, were used for immunohistochemical staining. The tumour samples used in this study include 74 adenocarcinoma, 36 squamous‐cell carcinoma and 7 large‐cell carcinoma samples. The histologic diagnosis was made according to the World Health Organization (WHO) classification guideline of lung cancer. The pathological diagnosis of tumour size, local invasion, lymph node involvement, distal metastasis and final disease stage were determined according to the American Joint Committee on Cancer (AJCC) TNM classification of lung cancer.

### 2.4 Statistics

The statistical significances of the experimental results were assessed by SPSS Statistics (IBM), with independent‐samples *T*‐test (two‐tailed), or Kaplan–Meier method with Mantel–Cox log‐rank test (for patient survival). *p* < 0.05 is considered significant.

Detailed experimental procedures are described in Appendix S1.

## 3 RESULTS

### 3.1 Knockdown of BMI1 inhibited the migration and invasion in LAC cells with active EGFR signalling

**FIGURE 1:**
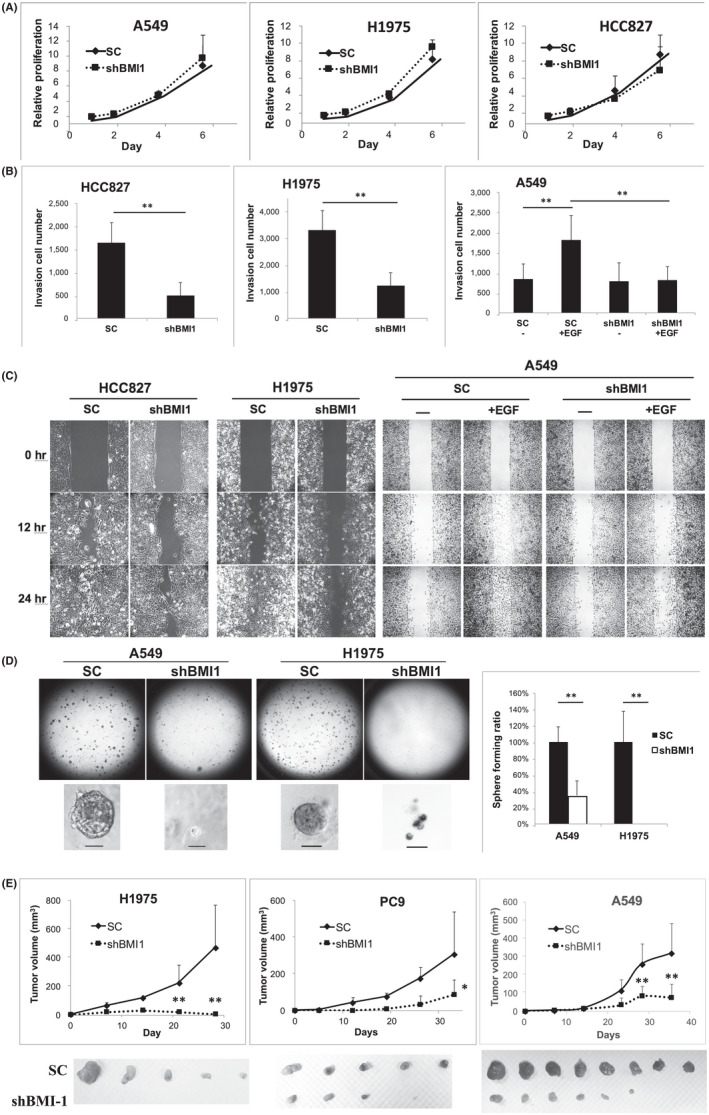
Knockdown of BMI‐1 inhibited the migration, invasion, spheroid formation and tumour formation of LAC cells. (A) LAC cells transduced with the SC or shBMI1 vectors were seeded in 96‐well plates and measured for cell viability on Days 1, 2, 4 and 6 after seeding. (B) LAC cells transduced with the SC or shBMI1 vectors were analysed for invasion activity in matrigel‐coated transwell, (for A549) with or without EGF treatment. (C) LAC cells transduced with the SC or shBMI1 vectors were analysed for migration activity by wound‐healing assay, (for A549) with or without EGF treatment. (D) LAC cells transduced with the SC or shBMI1 vectors were analysed for spheroid forming activity in serum‐free matrigel. Spheroids with a diameter >50 μm were counted and quantified. No sphere was found in H1975 shBMI1 group. The scare bar on the photo indicates 50 μm. (E) LAC cells transduced with the SC or shBMI1 vectors were subcutaneously injected into the flank region of mice, and the tumour sizes were measured weekly. At the end of experiment, tumours were resected and photographed. For H1975 shBMI1, no tumour mass was found subcutaneously after sacrificing the mice. The tiny tumour volumes measured on Days 14 and 21 should be the bodies of dead cancer cells and/or host inflammatory cells or fibroblasts, which were cleared later. In vitro experiments (A–D) were performed independently for 3 times. EGF, epidermal growth factor; SC, the scramble shRNA; shBMI1, the shRNA targeting BMI1; *p < 0.05; **p < 0.01

We firstly investigated how BMI1 may influence the biological functions of LAC cells. The proliferation rate of 3 LAC cell lines was measured. Unexpectedly, knockdown of BMI1 showed minimal impact on cell growth (Figure 1A). The invasion assay showed that knockdown of BMI1 significantly inhibited the invasive activity of HCC827 and H1975, but not A549 cells (shBMI1 vs. SC, Figure 1B). Since HCC827 and H1975 both contain mutant EGFR that auto‐phosphorylates and is constitutively active, while A549 contains wild‐type EGFR, we asked whether the activity of BMI1 is associate with EGFR state. The EGFR signalling was activated in A549 by EGF treatment. The results showed that EGF induced the invasion activity of A549 cells (SC + EGF vs. SC), while knockdown of BMI1 significantly blocked the EGF‐mediated invasion (shBMI1 + EGF vs. SC + EGF, Figure 1B). Likewise, knockdown of BMI1 inhibited the migration activity of H1975 and HCC827, but not A549 cells unless EGF was added (Figure 1C). The BMI1 knockdown efficiency was verified (Figure S1A). In summary, these results showed that knockdown of BMI1 inhibited migration and invasion of LAC cells with active EGFR.

### 3.2 Knockdown of BMI1 blocked the spheroid and tumour formations of LAC cells

We then tested whether BMI1 regulates the tumour initiation activity of LAC cells. H1975 and A549 cells both formed spheroids (>50 μm diameter) in serum‐free 3D matrix after 10–14 days of culture, while knockdown of BMI1 significantly blocked the spheroid formation (Figure 1D and Figure S1B). In mouse model, when LAC cells were subcutaneously implanted in nude mice, knockdown of BMI1 resulted in defective tumour formation in three cell lines tested (Figure 1E and Figure S1C). Although A549 contain wild‐type EGFR, knockdown of BMI1 still blocked spheroid and tumour formations (Figure 1D,E). It could be due to the existence of EGF in the medium for spheroid formation and in vivo, which activated EGFR signalling. Furthermore, knockdown of BMI1 reduced the side population (Figure S1D) and ALDH activity (Figure S1E) in A549 cells. Taken together, these results showed that BMI1 correlates with cancer stemness is required for tumour initiation in LAC cells.

### 3.3 BMI1 is positively regulated by EGFR and JNK signalling in LAC cells

**FIGURE 2:**
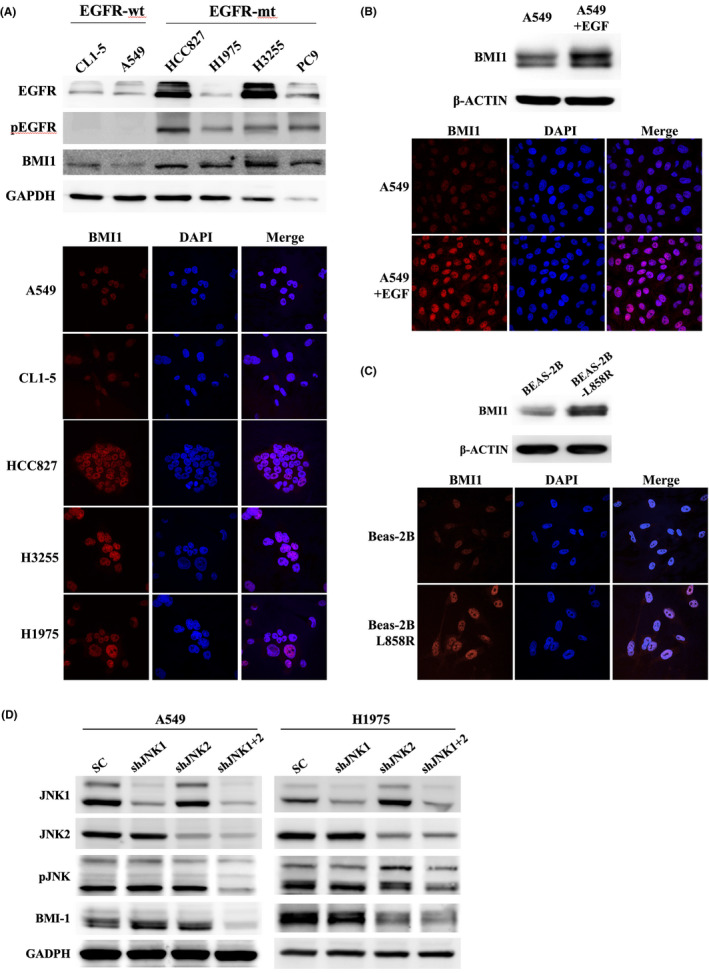
BMI‐1 was regulated through EGFR/JNK pathway in LAC cells. (A) Different LAC cell lines bearing wild‐type or mutant EGFRs (EGFR‐wt or EGFR‐mt) were detected for BMI1 expressions by Western blot or IF assay. (B) A549 cells were treated with EGF (100 ng/ml, 24 h) and detected for BMI1 expression by Western blot or IF assay. (C) BEAS‐2B cells were transduced with mutant EGFR (L858R) vector and detected for BMI1 expression by Western blot or IF assay. (D) LAC cells transduced with the SC, shJNK1, shJNK2 or shJNK1 + 2 vectors were detected for pJNK, JNK1, JNK2, BMI1 expressions. GAPDH or β‐ACTIN serve as loading control for Western blot. For IF, nuclei were counter‐stained by DAPI, and BMI1 signals (Dylight 549) were captured by the same exposure time in microscope for all the photos. SC, the scramble shRNA; shJNK1, the shRNA targeting JNK1; shJNK2, the shRNA targeting JNK2; shJNK1 + 2, the mixture of shJNK1 and shJNK2

We then tried to verify whether BMI1 expression is regulated through EGFR signalling in LAC cells. We firstly detected the endogenous BMI1 expression in different of LAC cell lines. The results showed that cell lines with mutant (active) EGFR generally expressed a higher BMI1 protein level as compared with those with wild‐type EGFR, either by Western blot or immunofluorescent (IF) assay (Figure 2A). Activation of EGFR signalling in A549 cells by EGF treatment also induced BMI1 expression (Figure 2B). Overexpression of mutant EGFR in BEAS‐2B (a non‐tumour human lung epithelial cell line) also induced BMI1 expression (Figure 2C). These data confirmed that EGFR signalling positively regulates BMI1 expression in LAC cells.

Since EGFR regulates multiple oncogenic pathways in lung cancer, we tried to clarify through which pathway it positively regulates BMI1. A549 cells were pretreated with different kinase inhibiters and then treated with EGF for 24 h. The results showed that only SP600125 (a JNK inhibitor) inhibited the EGF‐mediated BMI1 up‐regulation (Figure S1D). Treatment with SP600125 or Gefitinib (an EGFR inhibitor) in EGFR‐mutant cell lines also inhibited BMI1 expression (Figure S1E). To further confirm the association between JNK signalling and BMI1, specific knockdowns of JNKs using shRNAs were performed. We found that simultaneous knockdowns of both JNK1 and JNK2 stably inhibited pJNK and BMI1 expressions (Figure 2D), while knockdown of JNK1 or JNK2 alone frequently showed insufficient suppression to BMI1 (Figure 2D and data not shown). Knockdowns of JNK1 and JNK2 also blocked EGF‐mediated BMI1 upregulation (Figure S1F). In summary, our results showed that BMI1 expression was positively regulated by EGFR through JNK signalling in LAC cells.

### 3.4 EGFR/JNK regulated BMI1 protein stability through HUWE1 in LAC cells

**Figure 3:**
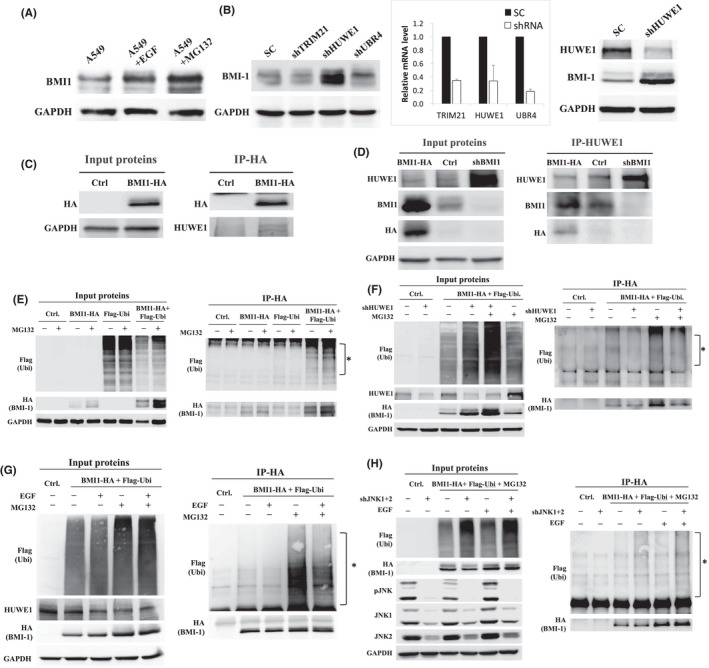
HUWE‐1 interacted with BMI1, and modulated BMI1 ubiquitination level and protein stability under the regulation of EGFR/JNK pathway. (A) A549 cells were treated with EGF (100 ng/ml, 24 h) or MG132 (10 μg/ml, 5 h) and detected for BMI1 expression by Western blot. (B) A549 cells transduced with the SC, shTRIM21, shHUWE1 or shUBR4 vectors were detected for BMI1 expression by Western blot (left), and the knockdown efficiencies were verified by Q‐PCR analysis (middle). Knockdown efficiency of shHUWE1 was also confirmed by Western blot (right). (C) A549 cells were transduced with Ctrl or BMI1‐HA vector (left), and the BMI1‐HA protein complex was precipitated from cell lysate by HA antibody, and analysed for HUWE1 expression by Western blot (right). (D) A549 cells were transduced with Ctrl or BMI1‐HA or shBMI1 vector (left), and the HUWE1 protein complex was precipitated from cell lysate by HUWE1 antibody, and analysed for BMI1 expression by Western blot (right). (E) A549 cells were transduced with Ctrl or BMI1‐HA, Flag‐Ubi or both BMI1‐HA and Flag‐Ubi vectors (left), and the BMI1 protein was precipitated from cell lysate by HA antibody, and analysed for the poly‐ubiquitination level of BMI1 by flag antibody (right). (F) A549 cells were transduced with Ctrl or BMI1‐HA and Flag‐Ubi, SC or shHUWE1 vectors (left), and the BMI1 protein was precipitated from cell lysate by HA antibody, and analysed for the poly‐ubiquitination level of BMI1 by flag antibody (right). (G) A549 cells were transduced with Ctrl or BMI1‐HA and Flag‐Ubi vectors and treated with EGF (left), and the BMI1 protein was precipitated from cell lysate by HA antibody, and analysed for the poly‐ubiquitination level of BMI1 by flag antibody (right). (H) A549 cells were transduced with Ctrl or BMI1‐HA and Flag‐Ubi, SC or shJNK1 + 2 vectors and treated with EGF (left), and the BMI1 protein was precipitated from cell lysate by HA antibody and analysed for the poly‐ubiquitination level of BMI1 by flag antibody (right). The ubiquitin signals (E–H) were quantified using ImageJ, and the relative intensities were indicated below the blotting. Ctrl, the control vector that expressed RFP; BMI1‐HA, the expression vector of BMI1 and HA‐tag fusion protein; Flag‐Ubi, the expression vector of Ubiquitin and Flag‐tag fusion protein; shHUWE1, the shRNA targeting HUWE1. * (for E to H) indicates the location of poly‐ubiquitinations. MG132 (10 μg/ml, 5 h) was used to stabilize the poly‐ubiquitinated proteins for co‐IP experiments

We then tried to clarify how EGFR/JNK regulates BMI1 expression. We firstly investigated the endogenous BMI1 mRNA level in different LAC cells. Surprisingly, BMI1 mRNA showed minimal variations among different cell lines (Figure S2A). Overexpression of mutant EGFR or knockdown of JNK1 and JNK2 did not change BMI1 mRNA level, either (Figure S2B,C). Furthermore, addition of EGF in A549 cells did not change BMI1 mRNA level for continuously 4 days, while BMI1 protein level was evidently enhanced (Figure S2D). In contrast, addition of MG132 (a proteasome inhibitor) mediated an evident increase in BMI1 protein level (Figure 3A). These results suggest that BMI1 is regulated mainly in a post‐transcriptional manner in LAC cells. The role of miRNA on BMI1 expression was also investigated by cloning BMI1‐3’UTR to the downstream of luciferase reporter gene. Nevertheless, the results showed that addition of EGF did not change the reporter gene expression with or without BMI1‐3’UTR (Figure S2E). While BMI1 has been reported to be regulated in protein stability by β‐TrCP E3‐ligase in breast cancer,^41^ our study of co‐immunoprecipitation (co‐IP) Western blot showed that BMI1 and β‐TrCP did not physically interact (Figure S2F).

To dissect the potential post‐transcriptional regulation of BMI1 in LAC cells, MASS spectrum was applied to analyse the proteins complexed to BMI1 (Figure S2F). The results identified 3 E3‐ligases in BMI1 co‐IP complex, which were TRIM21, HUWE1 and UBR4. We then found that only knockdown of HUWE1 significantly increased BMI1 protein level (Figure 3B). The physical interaction between BMI1 and HUWE1 was then confirmed by Western blot detecting HUWE1 in BMI1 co‐IP complex (Figure 3C). Likewise, both endogenous and exogenous (tagged with HA) BMI1 can be detected in HUWE1 co‐IP complex (Figure 3D). To confirm that BMI1 protein level was regulated through HUWE1‐mediated poly‐ubiquitination, HA‐BMI1 and flag‐Ubiquitin were co‐expressed. Firstly, we showed that flag‐Ubiquitin can be detected in HA‐BMI1 co‐IP complexes (Figure 3E), and knockdown of HUWE1 decreased the poly‐ubiquitination level of BMI1 (Figure 3F). We then found that addition of EGF decreased the poly‐ubiquitination level of BMI1 (Figure 3G). Finally, we showed that simultaneous knockdown of JNK1 and JNK2 increased the poly‐ubiquitination level of BMI1, and furthermore, addition of EGF cannot reduce the poly‐ubiquitination level elevated by knockdown of JNKs (Figure 3H), indicating that JNK signalling functions downstream of EGFR. In summary, these data showed that in LAC cells, BMI1 protein stability was regulated by HUWE1‐mediated poly‐ubiquitination, which induced the proteasomal degradation of BMI1. EGFR regulated BMI1 expression through JNK signalling, which inhibited the poly‐ubiquitination of BMI1.

### 3.5 JNK signalling and BMI1 regulated MCL1 expression through HUWE1

**Figure 4:**
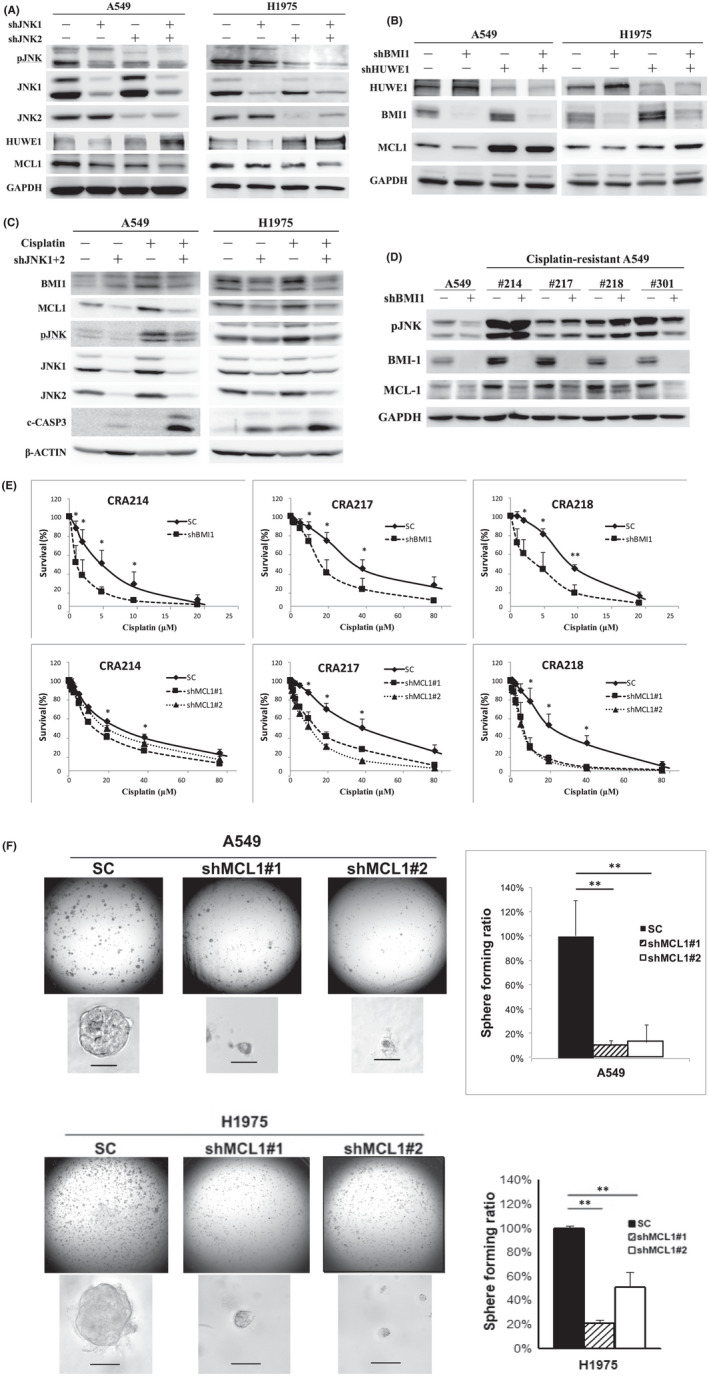
JNK signalling and BMI1 modulated MCL1 protein expression and promoted chemo‐resistance and spheroid formation in LAC cells. (A) LAC cells transduced with the SC, shBMI1, shHUWE1 or both shBMI1 and shHUWE1 vectors were detected HUWE1, BMI1 and MCL1 expressions by Western blot. (B) LAC cells transduced with the SC, shJNK1, shJNK2 or shJNK1 + 2 vectors were detected for HUWE1 and MCL1 expressions by Western blot. (C) LAC cells transduced with the SC or shJNK1 + 2 vectors and treated with cisplatin (10 μM, 24 h) were detected for BMI1, MCL1, pJNK and c‐CASP3 expressions by Western blot. (D) Parental A549 and 4 CRA clones were transduced with SC or shBMI1, and detected for pJNK, BMI1 and MCL1 expressions by Western blot. (E) CRA clones were transduced with SC, shBMI1 or shMCL1 vectors and analysed for cell viability after treated with different concentrations of Cisplatin for 4 days. (F) LAC cells transduced with the SC or shMCL1 vectors were analysed for spheroid forming activity in serum‐free matrigel. Spheroids with a diameter >50 μm were counted and quantified. The scare bar on the photo indicates 50 μm. CRA, cisplatin‐resistant A549; shMCL1, the shRNA targeting MCL1

We then asked whether JNK signalling modulates BMI1 poly‐ubiquitination level through regulating HUWE1 expression. We found that knockdown of JNK1 and JNK2 indeed enhanced HUWE1 expression (Figure 4A). MCL1, an BCL2 family member that regulates drug‐resistance and self‐renewal in leukaemia,^19^, ^20^, ^21^, ^24^ has been reported as a poly‐ubiquitination target of HUWE1.^42^ We found that enhanced HUWE1, after knockdowns of JNK1 and JNK2, was indeed accompanied by the reduction of MCL1 protein (Figure 4A). These data suggested that JNK signalling stabilized BMI1 and MCL1 proteins through suppressing HUWE1 expression. Nevertheless, it was also noticed that when overexpressing or silencing BMI1 in co‐IP experiments, BMI1 negatively regulated HUWE1 protein expression (Figure 3D,G, input panel). To confirm whether BMI1 also regulate HUWE1 expression, LAC cells were transduced with shBMI1, shHUWE1 or both. The results showed that knockdown of BMI1 indeed increased HUWE1 expression and decreased MCL1 expressions simultaneously (Figure 4B). Knockdown of HUWE1, as expected, increased both BMI1 and MCL1 expressions. Simultaneous knockdowns of BMI1 and HUWE1 still increased MCL1 expression, suggesting that HUWE1 regulated MCL1 protein stability downstream of BMI1 (Figure 4B). In contrast, overexpression of BMI1 or mutant EGFR both decreased HUWE1 and increase MCL1 expressions simultaneously (Figure S3A,B). JNK, BMI1 or HUWE1‐mediated regulation on MCL1 expression was post‐transcriptional, since knockdowns of these genes did not significantly change MCL1 mRNA level (Figure S3C). Likewise, BMI1 or JNK signalling did not regulate HUWE1 expression through mRNA transcription (Figure S3D,E). In summary, these data showed that JNK and BMI1 regulated MCL1 protein expression through HUWE1 in LAC cells.

### 3.6 JNK and BMI1 promoted the chemo‐resistance through MCL1 in LAC cells

Since MCL1 has been suggested as a major cause of resistance to radio‐ and chemo‐therapies,^20^, ^21^ we asked whether JNK and BMI1 induce the chemo‐resistance through MCL1 in LAC cells. Cells with or without the knockdown of JNKs were treated with Cisplatin for 24 h. The results showed that Cisplatin treatment induced the upregulations of pJNK, BMI1 and MCL1 (Figure 4C). Although most cells have not died within 24 h, cleaved CASPASE‐3 (c‐CASP3) can be detected. Knockdown of JNK1 and 2 blocked the up‐regulations of BMI1 and MCL1, and evidently enhanced the expression of Cisplatin‐induced c‐CASP3 (Figure 4C). The treatment with Doxorubicin showed the same results (Figure S3F). To further confirm the results, we selected the stable Cisplatin‐resistant A549 (CRA) clones by long‐term and chronic cisplatin treatments. Western blot analysis showed that in 4 clones tested, all CRA cells showed enhanced pJNK, BMI1 and MCL1 as compared with parental A549 (Figure 4D). Likewise, knockdown of BMI1 suppressed MCL1 protein expression (Figure 4D), but not mRNA level (Figure S3G). Knockdown of either BMI1 or MCL1 significantly sensitize LAC cells to Cisplatin treatment (Figure 4E).

Finally, since MCL1 plays an important role in the survival and self‐renewal in both malignant lymphocytes and haematopoietic stem cells,^19^, ^24^ we tested whether it is also indispensable for self‐renewal in LAC cells. The results showed that knockdown of MCL1, like BMI1, did not inhibit cell growth in regular passages in culture dish, but blocked the spheroid forming ability (Figure 4F and Figure S3H). Altogether, these results suggested that through the stress‐activated kinase JNK, LAC cells can stabilize BMI1 and MCL1 protein expressions, and desensitize to chemotherapy‐induced apoptosis. Knockdown of BMI1 or MCL1 significantly sensitized LAC cells to chemotherapy agent.

### 3.7 Knockdown of BMI1 did not modulate the expressions of PTEN, pAKT or p16INK4A in LAC cells

BMI1 has been documented to regulate a number of genes in cancers, among which PTEN, phosh‐AKT (pAKT) and p16INK4A were highly associated with oncogenesis. We thus verified whether BMI1 regulates the expression of these genes in LAC cells. Unexpectedly, our results did not suggest that knockdown of BMI1 can significantly and reproducibly downregulate pAKT via upregulation of PTEN in LAC cell lines tested (Figure S4A). Knockdown of BMI1 did not significantly enhance p16INK4A in mRNA or protein level, either (Figure S4B,C). Expressions of EMT‐associated factors, such as SNAIL, SLUG, TWIST, were also investigated. However, protein expressions of these genes were not changed after knockdown of BMI1 (Figure S4D). These combined results suggest that BMI1‐mediated oncogenesis can be variable in different types of tumour.

### 3.8 The clinical significance of BMI1 and MCL1 in NSCLC patients

**Figure 5:**
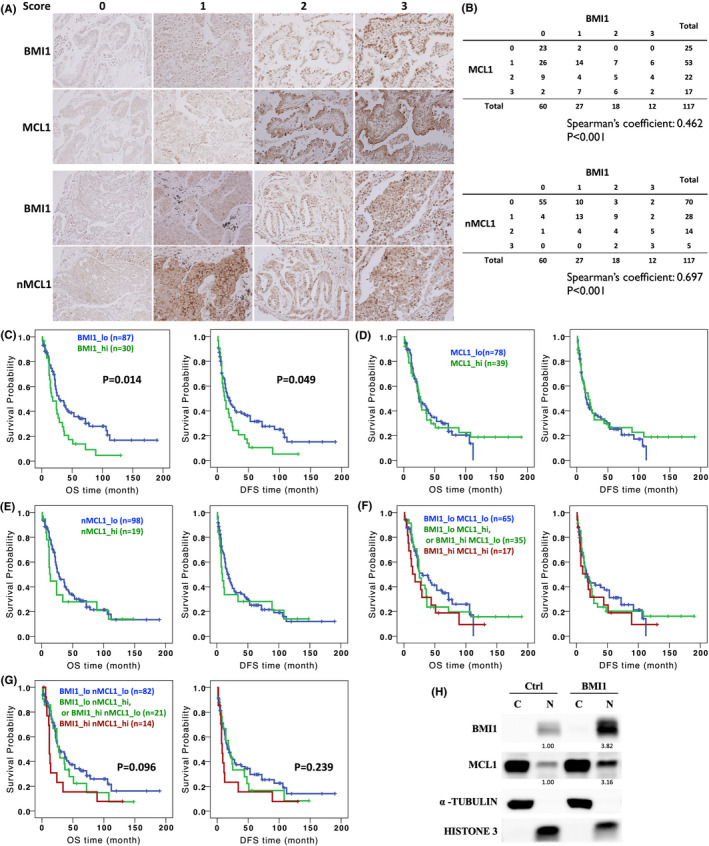
Clinical significance of BMI‐1 and nMCL‐1 in NSCLC patients. (A) Totally 117 lung cancer samples (74 adenocarcinoma, 36 squamous‐cell carcinoma and 7 large‐cell carcinoma samples) were subjected to IHC staining, and the results were classified into score 0–3, according to the intensity and the ratio of positively stained cells. For MCL1, overall MCL‐1 (indicated as ‘MCL1’, scoring the total intensity of MCL1 in tumour cells) and nuclear MCL‐1 (indicated as ‘nMCL1,’, scoring the intensity of MCL1 only in nucleus) were independently scored. Two sets of patient samples were illustrated on the Figure. (B) The correlation between BMI1 and MCL1, or BMI1 and nMCL1 was analysed by Spearman's rank correlation test. Patients were grouped into (C) BMI1_lo and BMI1_hi, (D) MCL1_lo and MCL1_hi, (E) nMCL1_lo and nMCL1_hi (F) BMI1_lo MCL1_lo, BMI1_lo MCL1_hi or BMI1_hi MCL1_lo, and BMI1_hi MCL1_hi, (G) BMI1_lo nMCL1_lo, BMI1_lo nMCL1_hi or BMI1_hi nMCL1_lo, and BMI1_hi nMCL1_hi and analysed for OS and DFS. (H) A549 cells were transduced with BMI1 expression vector and analysed for MCL1 expressions in nucleus and cytosol by Western blot. αTUBULIN and HISTONE3 served as loading control for cytosolic and nuclear proteins, respectively. The signals of nuclear BMI1/MCL1 were quantified with ImageJ, and the relative intensities were indicated below the blotting. The score of IHC staining: 0, no staining; 1, weak staining; 2, moderate staining; 3, strong staining. BMI1_lo, BMI1 scores 0 and 1; BMI1_hi, BMI1 scores 2 and 3; MCL1_lo, MCL1 scores 0 and 1; MCL1_hi, MCL1 scores 2 and 3; nMCL1_lo, nMCL1 scores 0 and 1; nMCL1_hi, nMCL1 scores 2 and 3. OS, overall survival; DFS, disease‐free survival

We then investigated the clinical significance of BMI1 and MCL1 in lung cancer patients. Totally 117 lung cancer samples were subjected to immunohistochemical (IHC) staining, and the results were classified into score 0 to 3, according to the intensity and the ratio of positively stained cells (Figure 5A). BMI1 was positively stained in 48.7% (57/117), and MCL1 in 78.6% (92/117) of lung tumour samples. Unexpectedly, MCL1 showed evident nuclear localization in a number of samples. Therefore, overall MCL1 (indicated as ‘MCL1’, scoring the total intensity of MCL1 in tumour cells) and nuclear MCL1 (indicated as ‘nMCL1’, scoring the intensity of MCL1 only in nucleus) were independently scored. The correlation analyses showed that BMI1 expression was significantly correlated to both MCL1 and nMCL1, while the coefficient value of nMCL1 was greater than MCL1 (0.697 vs. 0.462) (Figure 5B). For survival analysis, the staining intensities were divided into ‘low’ and ‘high’, which include scores 0 and 1 (low), and scores 2 and 3 (high), respectively. Patients with high expression of BMI1 (BMI1_hi) was significantly correlated with a poor survival as compared with those with low expression of BMI1 (BMI1_lo), either in terms of overall survival (OS) time of disease‐free survival (DFS) (Figure 5C). The expression of MCL1 or nMCL1, however, was not correlated with survival (Figure 5D,E). We then asked whether simultaneously high expressions of BMI1 and MCL1 would correlate with a worse survival. The patients were grouped in 3: BMI1_hi MCL1_hi, BMI1_hi MCL1_lo or BMI1_lo MCL1_hi and BMI1_lo MCL1_lo. The results showed that BMI1_hi MCL1_hi was still not correlated with patient survival (Figure 5F). However, BMI1_hi nMCL1_hi was associated with a worse survival as compared with other groups, although the difference did not reach the statistical significance (*p* = 0.096 for OS and *p* = 0.239 for DFS, respectively), presumably due to the limited sample numbers (Figure 5G). Finally, we tested whether BMI1 can promote the nuclear expression of MCL1 in cell model. The immunofluorescent microscopy showed that endogenous MCL1 distributed mostly in cytosol, and partially in nucleus (Figure S5). Knockdown of BMI1 decreased both the total and nuclear MCL1 expression (Figure S5A). In accordance, overexpression of BMI1 enhanced the nuclear MCL1 (Figure S5B). Western blot analysis confirmed that MCL1 in nuclear fraction was increased after BMI1 overexpression (Figure 5H). In summary, these data showed that the protein expression of BMI1 is significantly correlated with nuclear MCL1 in lung tumours, and the patients with simultaneous high expressions of BMI1 and nuclear MCL1 may correlate with a poor prognosis.

### 3.9 The development of therapeutic agents targeting BMI1 and MCL1 for LAC treatment

**Figure 6:**
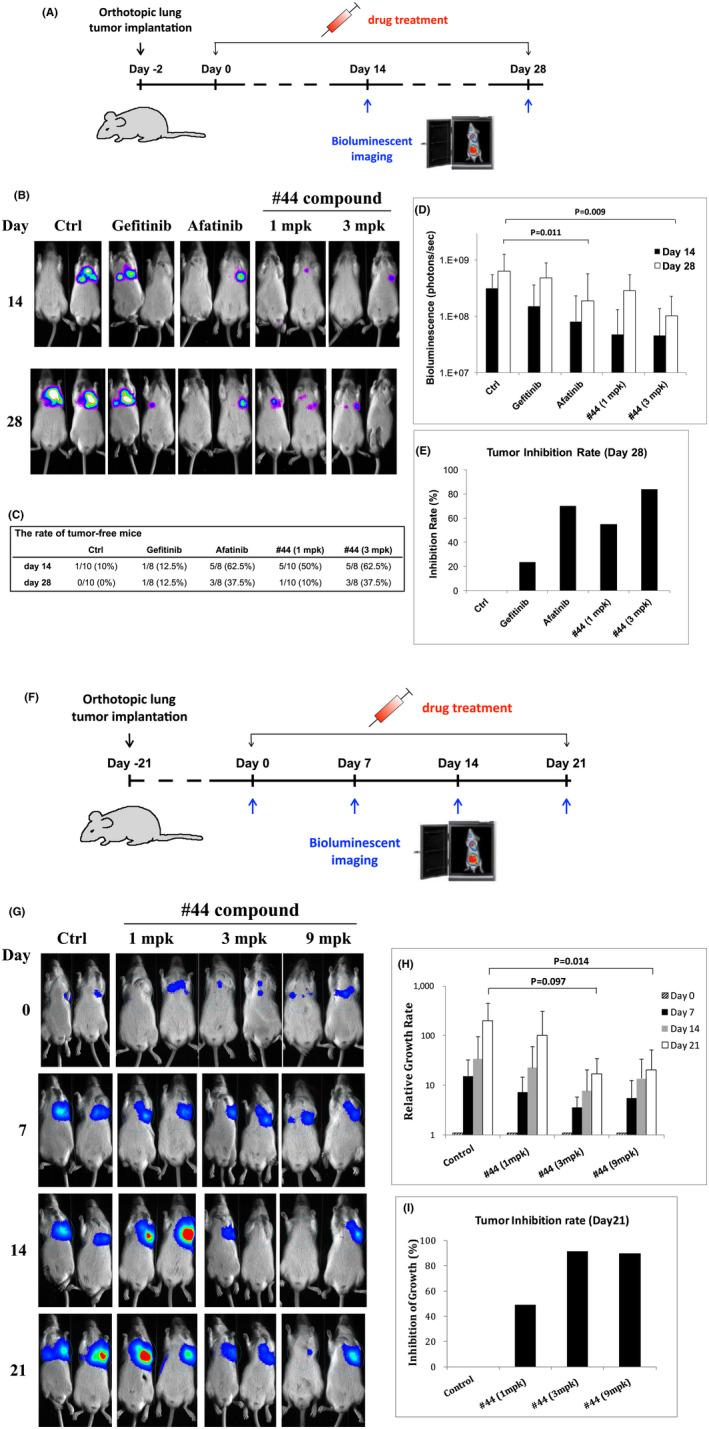
Development of a novel small‐molecule that targets BMI1 and MCL1 for LAC treatment. (A) Mice were orthotopically implanted with H1975‐luc cells (10^6^ cells/mouse). On Day 0 (defined as 2 days after tumour implantation), mice were started to receive drug treatments for 4 weeks (5 times/week), and the tumour formations were followed by non‐invasive imaging on Days 14 and 28. (B) Illustrations of the imaging results of some mice in each group. (C) The rate of tumour‐free mice in each group on Days 14 and 28. (D) The quantification of bioluminescent intensities of the mice in each group. *N* = 10 for Ctrl and #44 1 mpk and *n* = 8 for other groups. (E) The tumour inhibition rate in of each group on Day 28. (F) Mice were orthotopically implanted with H1975‐luc cells (10^6^ cells/mouse). On Day 0 (defined as 3 weeks after tumour implantation), mice were imaged and started to receive drug treatments for 3 weeks (5 times/week). The tumour growths were followed by non‐invasive imaging weekly. (G) Illustrations of the imaging results of some mice in each group. (H) The relative growth rates of the mice in each group were averaged and presented. *N* = 7 for Ctrl and #44 1 mpk, and *n* = 8 for other groups. (I) The tumour inhibition rate in of each group on Day 21. H1975‐luc, the H1975 cells that were stably transduced with a luciferase expression vector. mpk, mg per kg of body weight.Compound #44 was administrated by IV injection through tail vein, 5 times/7 days, with the doses indicated on the figure. Gefitinib and Afatinib were administrated orally, 5 times/7 days, with the dose of 20 mpk

To develop therapeutic agent targeting BMI‐1/MCL‐1, we applied the small‐molecule compound library and identified a compound, Lisuride (Figure S6A), which was predicted to mediate similar gene expression signatures such as knockdown of BMI‐1 in LAC cells. The preliminary tests confirmed that Lisuride inhibited BMI‐1 expression and H1975 spheroid formation in a dose‐dependent manner (Figure S6B,C). Since Lisuride is an antiparkinson agent, chemical modification was applied to reduce its Blood–Brain barrier penetration ability and to improve its water‐solubility. The closed ring of the main core was opened to reduce its planarity, and several high polarity groups were introduced to reduce its lipophilicity hydrophobicity (Figure S6D). More than 100 derivatives of Lisuride were synthesized and tested for anti‐BMI1/MCL1 efficacy in vitro (Figure S6E,F). The derivatives showing potent anti‐BMI1/MCL1 effect, such as compounds #43–45, were selected for further in vivo anti‐tumour test (Figure S6G), in which compound #44 (named as BI‐44 in this study) showed significant anti‐tumour growth effect. The efficacy of BI‐44 was further examined in two mouse models. First, BI‐44 was started to be administrated 2 days after orthotopic lung tumour implantation (Figure 6A), and the tumour formations were evaluated via non‐invasive bioluminescent imaging (Figure 6B). Clinical drugs Gefitinib (1st‐generation TKI, which does not target EGFR T790M) and Afatinib (2nd‐generation TKI, which can target EGFR T790M) were used as negative and positive controls, respectively, since H1975 contains a T790M mutation on EGFR. The results showed that the group treated with BI‐44 in the dose of 3 mg per kg of body weight (3 mpk) had a tumour‐free rate of 62.5% (5/8) and 37.5% (3/8) on Days 14 and 28, respectively, corresponding to the group treated with Afatinib (Figure 6C). The quantification of bioluminescent imaging showed significantly reduced tumour signals in mice treated with BI‐44 (3 mpk) or Afatinib (Figure 6D), with tumour inhibition rates around 80% as compared to control (Figure 6E). In the second model, BI‐44 was started to be administrated 3 weeks after tumour implantation when all the mice contained defined tumour signals in lungs, and the tumour growth rates were followed for 3 weeks (Figure 6F,G). The results showed that BI‐44 significantly inhibited tumour growth in a dose‐dependent manner (Figure 6H). The inhibition rates of 3 and 9 mpk on Day 21 were around 90% (Figure 6I). All treatments in both models did not change mice body weights during the experiments (Figure S6H,I). Finally, although our preliminary analysis showed that BI‐44 bound to BMI1 dimer and showed little structural similarity to known kinase inhibitors (data not shown), the potential off‐target inhibitions of BI‐44 to kinases downstream of EGFR were also tested. The results confirmed that BI‐44 did not inhibit the phosphorylation levels of AKT, ERK, JAK or JNK (Figure S6J). In summary, our study showed that the novel small‐molecule BI‐44 developed in this study can efficiently repress BMI‐1 and MCL‐1 protein expressions and inhibit lung tumour formation and progression in pre‐clinical model.

## 4 DISCUSSION

Drug resistance and tumour recurrence remain to be the major challenges for lung cancer treatment in clinic. CSC model is a possible explanation of tumour heterogeneity, in which the cells acquiring stemness properties maintain and repopulate the tumour tissue, driving tumour progression, metastasis, drug resistance and the relapse.^4^, ^5^ It has gradually become clear that CSCs do not necessarily be rare and/or quiescent, and the CSC hierarchies may not be rigid, in which CSCs and non‐CSCs are plastic and capable of undergoing phenotypic transitions in response to environmental stimuli.^5^ CSC phenotype can be driven by diverse signalling pathways, such as ERK and AKT signalling. In contrast, JNK signalling is relatively less studied in CSC phenotype regulation. The current study focused on the role of stemness factor BMI1 in lung cancer, and its crosstalk with EGFR through JNK‐mediated protein stability regulations.

In this study, we found that knockdown of BMI1 blocked LAC cell migration, invasion, chemo‐resistance and tumour initiation in vitro and in vivo (Figures 1 and 4E), confirming the previous recognition of BMI1 as a stemness regulator in cancer.^8^, ^9^, ^10^, ^11^ Accordingly, targeting BMI1 with BI‐44 suppressed LAC tumour formation and progression in pre‐clinical model (Figure 6). These combined results suggest that LAC tumour can be reliant on BMI1 to sustain the survival and progression in vivo, and targeting BMI1 is a promising approach for LAC treatment. An important concern of using BMI1 inhibitor could be the toxicity to normal tissue stem cells such as those in lung and intestine.^7^, ^8^, ^43^ Of note, the transient inhibition of BMI1 in tumour with small molecules would not be comparable to the genetic ablation of BMI1 in tissue cells in transgenic mice as presented in those studies. In our study, no changes in animal body weight or severe adverse effects were observed during experiments (Figure S6H,I), suggesting that the dose of small molecules used to lower tumour burden would not noticeably affect normal tissue.

While the functional role BMI1 in oncogenesis has been widely studied,^7^, ^8^, ^9^, ^10^, ^11^, ^12^, ^44^, ^45^ much less is known about how it is regulated. In this study, we showed that BMI1 is regulated through EGFR and JNK signallings in LAC cells. Activation of JNK, either through EGFR or chemotherapy agents, can stimulate BMI1 expression (Figures 2 and 4C,D, Figures S1 and S3F). Since JNK is a stress‐activated protein kinase, our results suggest that LAC cells could acquire an CSC‐like phenotype through the activation of JNK, either by oncogene activation (e.g., EGFR mutation) or environmental stimuli (e.g., genotoxic agents). More specifically, JNK regulates BMI1 through stabilizing the protein expression but not mRNA transcription. The E3‐ligase HUWE1 physically interacts with BMI1, induces the poly‐ubiquitination and proteasomal degradation of BMI1 in LAC cells (Figure 3 and S2F). Treatment of EGF or knockdown of HUWE1 reduced the poly‐ubiquitination level of BMI1 (Figure 3F,G). In contrast, knockdown of JNKs increased the poly‐ubiquitination level of BMI1, even in the presence of EGF (Figure 3H), confirming the key role of JNK signalling in modulating the BMI1 expression in LAC. JNK signalling modulates BMI1 expression through suppressing HUWE1 protein expression (Figure 4A). Interestingly, up‐regulation of BMI1 also suppressed HUWE1 protein expression (Figure 3D,G and Figure S3A), which could further stabilize itself and MCL1 expressions. Although BMI1 is a key regulatory component of the PRC1, which modulates chromatin structure and thereby regulates the transcription of a number of important genes, it is unlikely that HUWE1 is regulated by BMI1 through the known PRC‐related mechanism since the mRNA expression of HUWE1 was not significantly changed after overexpression or knockdown of BMI1 (Figure S3D,E). How BMI1 and JNK signalling regulate HUWE1 expression post‐transcriptionally remains further studies to clarify. Collectively, these combined data suggest that JNK signalling plays a key role to link oncogenic pathway or environment stress to cancer stemness, regulating self‐renewal and chemo‐resistance through modulation of protein stability of BMI1 in LAC cells.

Previous studies have shown that BMI1 regulates a variety of gene expressions and signalling pathways in different types of tissue stem cell or cancer.^9^, ^11^ Although the tumour‐suppressor p16INK4A is a well‐documented target of BMI1‐induced gene silencing,^9^, ^11^ knockdown of BMI1 did not significantly change p16INK4A expression either in mRNA or protein level in our study (Figure S4B,C). This result suggests that p16INK4A expression would be unconnected to BMI1, or 16INK4A expression has been dominantly suppressed through other mechanisms in LAC cells. Similarly, knockdown of BMI1 did not significantly change the expression of PTEN, pAKT or other EMT regulators including SNAIL, SLUG, TWIST in our LAC cell lines tested (Figure S4A,D). Although the results did not exclude the possibility that BMI1 may still cooperate with these factors in different manners, such as protein–protein interaction with TWIST,^46^ it would suggest that the oncogenic role of BMI1 is context‐dependent in different types of cancer. In this study, we showed that MCL1 is a key factor downstream of BMI1 in LAC cells. MCL1 protein expression can be stabilized by EGFR/JNK and/or BMI1‐mediated suppression of HUWE1 expression, which promotes chemo‐resistance and regulates self‐renewal (Figure 4). Although MCL1 is best‐known for its role in anti‐apoptosis,^19^, ^20^, ^21^ it is also a key regulator of self‐renewal in both malignant lymphocytes and haematopoietic stem cells.^19^, ^24^ The functional role of MCL1 in cancer stemness other than leukaemia yet has been less investigated. Recent studies showed that MCL‐1 can reside at the mitochondrial matrix in pluripotent stem cells and regulate the pluripotency through mitochondrial dynamics.^47^ Furthermore, MCL1 is highly expressed in embryonic stem cells, and inhibition of MCL1 selectively kills embryonic stem cells and induced‐pluripotent stem cells.^48^, ^49^ The exact role that MCL1 plays in tumour initiation in LAC cells remains unclear, and deserves further studies to clarify.

An interesting finding of this study is that in clinical samples, BMI1 is highly correlated with nMCL1, and the samples with simultaneous high expressions of BMI1 and nMCL1 tends to have a poor survival (Figure 5). The exact role of nMCL1 remains unclear. Previous studies suggested that the nuclear localization of MCL1 is associated with DNA damage response.^28^, ^29^, ^30^, ^31^ A recent study showed that nMCL1 promotes homologous recombination‐dependent DNA double‐strand break repair.^50^ The nuclear localization of MCL1 is suggested to be regulated through its N‐terminal domain since an earlier study showed that deletion of the first 79 amino acids increased the nuclear expression and the anti‐proliferative activity of MCL1.^51^ In this study, although we showed that BMI1 can promote nMCL1 expressions (Figure 5H and Figure S5), protein co‐IP did not find the physical interaction between BMI1 and MCL1 (Figure S2F). How BMI1 regulates the nuclear localization of MCL1, and whether nMCL1 cooperates with BMI1 to modulate chemo‐resistance and self‐renewal in LAC deserve future studies to clarify.

## 5 CONCLUSIONS

In summary, this study shows that in lung cancer, JNK signalling is a link between oncogenic pathway or environment stress to cancer stemness (Figure S7). The activation of JNK, either by EGFR or chemotherapy agent, stabilizes BMI1 and MCL1 protein expressions through suppressing HUWE1 expression, which then promote tumour initiation and chemo‐resistance. The expression of BMI1 is positively correlated with MCL1 in clinical lung tumour samples, and the high level of BMI1 is correlated with poor survival. The novel small‐molecule BI‐44 developed in this study effectively suppressed BMI1/MCL1 expressions and inhibited tumour initiation and progression in preclinical models. Targeting BMI1/MCL1 thus provides a new and promising therapeutic approach for the treatment of lung cancer.

## AUTHOR CONTRIBUTIONS

**Erh‐Hsuan Lin:** Conceptualization (lead); data curation (equal); formal analysis (equal); investigation (equal); methodology (equal); project administration (equal); supervision (equal); writing – original draft (lead); writing – review and editing (lead). **Jhen‐Wei Hsu:** Data curation (equal); formal analysis (equal); investigation (equal); validation (equal). **Ting‐Fang Lee:** Writing – review and editing (equal). **Chiung‐Fang Hsu:** Data curation (equal); formal analysis (equal); investigation (equal). **Tsung‐Hsien Lin:** Data curation (equal); formal analysis (equal); investigation (equal). **Yi‐Hua Jan:** Formal analysis (equal); investigation (equal). **Hsiang‐Yi Chang:** Data curation (equal); formal analysis (equal); investigation (equal). **Chun‐Ming Cheng:** Data curation (equal); formal analysis (equal). **Hui‐Jan Hsu:** Data curation (equal); methodology (equal). **Wei‐Wei Chen:** Data curation (equal); methodology (equal). **Bo‐Hung Chen:** Data curation (equal); formal analysis (equal). **Hsing‐Fang Tsai:** Data curation (equal); formal analysis (equal). **Jung‐Jung Li:** Data curation (equal); formal analysis (equal); investigation (equal). **Chi‐Ying Huang:** Resources (supporting); supervision (supporting). **Shih‐Hsien Chuang:** Data curation (equal); methodology (equal); resources (supporting). **Jia‐Ming Chang:** Resources (supporting); supervision (supporting). **Michael Hsiao:** Resources (supporting); supervision (supporting). **Cheng‐Wen Wu:** Funding acquisition (supporting); investigation (supporting); resources (supporting); supervision (supporting).

## CONFLICT OF INTEREST

National Yang‐Ming University and Development Center of Biotechnology have a patent (PCT/US2018/030300) concerning the therapeutic compounds used in this study, where Cheng‐Wen Wu, Erh‐Hsuan Lin, Chi‐Ying Huang, Hui‐Jan Hsu, Wei‐Wei Chen, Shih‐Hsien Chuang and Jia‐Ming Chang are also the authors of the patent.

## Supporting information


Appendix S1

## Data Availability

Availability of data and materials The datasets used and/or analysed during the current study are available from the corresponding author on reasonable request.
