# Supplementary material for: Targeting cancer stemness mediated by BMI1 and MCL1 for non‐small cell lung cancer treatment
Source: J Cell Mol Med. 2022 Jul 6;26(15):4305–21. doi: 10.1111/jcmm.17453 (PMC9401641; doi:10.1111/jcmm.17453)
Supplement: Supplementary file 1 — Appendix S1 [file JCMM-26-4305-s001.docx]

# Supplementary Materials and Methods

## Cell culture

Lung adenocarcinoma (LAC) cell lines, including A549 (ATCC CCL-185), H1975 (ATCC CRL-5908), HCC827 (ATCC CRL-2868), H3255 (ATCC CRL-2882), CL1-5, and PC9 were cultured in RPMI medium supplemented with 10% fetal bovine serum (FBS) and 1% penicillin/streptomycin. Human non-tumor lung epithelial cell line BEAS-2B (ATCC CRL-9609) was cultured in F12 medium containing 4% FBS, 1% penicillin/streptomycin and supplemented with 0.1 mM non-essential amino acids, 500 ng/ml hydrocortisone, 1x ITS (Insulin-Transferrin-Selenium), and 10 ng/ml EGF. HEK293T cells were cultured in Dulbecco’s modified Eagle’s medium (DMEM) medium supplemented with 10% FBS and 1% penicillin/streptomycin. All cells were cultured at 37^o^C and 5% CO_2_ incubator. Chemicals used are listed in Table S5.

## Generation of Cisplatin-Resistant A549 (CRA) cell lines

CRA cells were produced by long-term treatment with gradually increased concentrations of cisplatin (0.1 μM for 1 week, 0.5 μM for 1 week, 1 μM for 2 weeks, 5 μM for 2 weeks, 10 μM for 2 weeks), and sub-cultured to select single-cell clones that can grow up in the medium containing 10 μM of cisplatin in 4 weeks.

## Plasmids

The mutant EGFR (L858R) expression vector pBabe-puro-EGFR(L858R) was purchased from Addgene (#11012, Addgene, Watertown, MA, USA). BMI1-HA cDNA sequence was obtained from pT3-EF1a-BMI1 (Addgene #31783), which was amplified by PCR and cloned into pLAS3w.Ppuro vector (National RNAi Core Facility) via NheI site. The expression vector of Flag-Ubiquitin fusion protein was kindly provided by Dr. Hsiu-Ming Shih (IBMS, Academia Sinica). The Luciferase expression vector pGL3-promoter was purchased from Promega (E1761). The RFP Lentiviral vectors pLAS3w.RFP-C.Ppuro was purchased from National RNAi Core Facility, which serves as control (Ctrl) plasmid in the experiments of overexpression.

Δ8.9, pVSV-G, and all pLKO.1 shRNA vectors used in the study were purchased from National RNAi Core Facility (Academia Sinica). The target sequences and corresponding genes are listed on Table S3.

## Lentiviral vector production

Twenty-four hours before transfection, HEK293T cells were seeded on a 10-cm culture dish to reach 60 – 70% confluency. The day of transfection, 10 μg of lentiviral vector, 9 μg of Δ8.9 plasmid, and 2.5 μg of pVSV-G were co-transfected into HEK293T, using jetPEI transfection reagent according to the manufacturer’s instructions. The transfection medium was replaced with fresh culture medium 24 h later. The medium containing virus was collected 48 h post-transfection, and stored at -80℃ before use.

## Cell proliferation assay

The day before experiment, LAC cells were seeded in a 96-well plate (1000 cells/well), and the cell viabilities at different time points were detected using AlarmaBlue reagent (Table S5), measured at 560nm (excitation) and 590 nm (emission) according to the manufacturer’s instructions. The cell viability measured on day 1 was set as 1, and the relative viabilities at different time points were presented.

## Cell survival curve assay

The day before experiment, cells were seeded in 96-well plates (1000 cells/well). The next day (day 0), fresh mediums containing different concentrations of cisplatin were added into wells and incubated for 72 h. The cell viability was measured using AlarmaBlue.

## Cell colony forming assay

LAC cells were seeded in 6-well plates (1000 cells/well), and cultured for 10 – 14 days. The culture medium was replaced with fresh one every 3 – 4 days. At the end of experiment, cells were washed twice with PBS and fixed with 4% paraformaldehyde for 15 min, and then stained with 0.1% crystal violet for 30 min, and de-stained with distilled water.

## Invasion assay

Non-transparent trans-wells (FluoroBlok) were coated with 7 μl of matrigel that was 1:1 diluted with RPMI. LAC cells (5 x 10^4^ cell/well) cells were suspended in 200 μl of culture medium and seeded on the top of matrigel, and incubated for 18 h. Cells were then washed twice with PBS, fixed with 100% methanol for 30 min, washed twice with PBS, and then stained with Hoechst for 5 min. The invasive cells were imaged using fluorescent microscopy and quantified with ImageJ software. Chemicals used are listed in Table S5.

## In vitro tumor spheroid formation assay

Before experiment, 96-well plates were coated with 0.7% agarose (50 μl/well). LAC cells were suspended in Matrigel and loaded on the top of agarose layer (2000 cells/30 μl/well). To avoid pipetting error, cell/matrigel mixtures for 6 replicates were prepared in one tube, and loaded to 5 wells. DMEM/F12 medium containing 1x N2 Supplement, 50 ng/ml of EGF, and 60 ng/ml of FGF was added to the wells (200 μl/well), and refreshed every 3 – 4 days. Two weeks later, spheroids formed in matrigel were examined and imaged under microscope. Only spheroids with the diameter >50 μm were counted and quantified.

## RNA extraction and Quantitative PCR (qPCR)

RNA was extracted from the clear supernatant using GENEzol Reagent (Maestrogen) according to the manufacturer’s instructions, and then quantified and stored in Rnase-free water at -80°C until use. Reverse transcription was performed using Superscript III reverse transcriptase, and the cDNAs were subjected to real-time PCR on a 96-well/plate Lightcycler 480 machine (Roche, core facility of IBMS, Academia Sinica), combined with SybrGreen MasterMix or Universal Probe Library system (Roche Applied Science). Relative gene expression was analyzed using the 2–ΔΔCT method, with 18S rRNA as reference transcript. Primer sequences designed to detect specific genes are listed on Table S4. Chemicals are listed in Table S5.

## Protein extraction and Western-blotting

Cells were washed with PBS and lysed in RIPA buffer (1% NP-40, 10 mM Tris-HCl pH7.4, 150 mM NaCl, 1 mM DTT, 5 mM EDTA and 1 mM PMSF) containing PhosSTOP (protease and phosphatase inhibitor cocktails), and sonicated with Water Sonication (Bioruptor UCD-200, Diagenode). Protein concentration was determined using DC Protein Assay Reagent. Protein samples were resolved in 8% or 10% SDS-PAGE and transferred to PVDF membrane. The membrane was blocked with 2.5% BSA in TBST (10 mM Tris-HCl pH7.4, 150 mM NaCl, 0.1% Tween 20) for 1 h at room temperature, and then hybridized with first antibodies at 4^o^C overnight. After wash, the membrane was hybridized with secondary antibodies at room temperature for 1 h. After wash, the protein signals were detected using ImageQuant LAS-4000 after incubation with Immobilon Western Chemiluminescent HRP Substrate or SuperSignal West Pico Chemiluminescent Substrate. If necessary, the intensity of each band was quantified using ImageJ, normalized by internal control, and shown as relative intensity to control group. The antibodies and the chemicals used are listed in Table S1, S2, and Table S5, respectively.

## Nuclear and cytosolic protein extraction

Cells were lysed in hypotonic buffer (20 mM N-2-hydroxyethylpiperazine-N’-2-ethanesulfonic acid, 10 mM KCl, 2mM MgCl_2_, 0.5% NP40, 1 mM Na_3_VO_4_, 1 mM phenylmethylsulfonyl uoride and 1 mM dithiothreitol, pH 7.0). After 2 centrifugations (1,000 RPM for 5min, and 13,000 RPM for 5 min), the supernatant (containing the cytosolic protein fraction) was collected, and the pellet (containing the nuclear fraction) was washed 3 times with the hypotonic buffer and then lysed in NP40 lysis buffer (50 mM N-2- hydroxyethylpiperazine-N’-2-ethanesulfonic acid, 150 mM NaCl, 1% NP40, 5 mM ethylenediaminetetraacetic acid, 1 mM Na3VO4, 1 mM phenyl-methylsulfonyl uoride, and 1 mM dithiothreitol, pH 7.4). After sonication and centrifugation (13,000 RPM for 20min), the supernatant of the nuclear fraction (containing the nuclear protein) was collected.

## Protein co-immunoprecipitation (co-IP)

Protein sample (500 μg protein/200 μl RIPA/tube) derived from cell lysate was incubated with 1 μg of primary antibody overnight at 4℃, followed by incubation with Dynabeads for 1 h at room temperature (RT) to capture the immunocomplex. After wash, the immunocomplex was eluted by boiling in 1× sodium dodecyl sulfate sample buffer (50mM Tris, pH 6.8, 2% sodium dodecyl sulfate, 10% glycerol, 5% β-mercaptoethanol and 0.001% bromophenol blue) for SDS-PAGE and western-blot. Chemicals and materials used are listed in Table S5.

## Immunofluorescent (IF) staining

Cells grown on the chamber slides were fixed with 4% PFA for 10 min at RT, washed with PBS, permeabilized with 0.1% Triton X-100 for 10 min at RT, washed with PBS, and blocked with 5% milk in PBS. The cells were then incubated with primary antibody overnight at 4℃, washed with PBS, and incubated with secondary antibody conjugated with proper fluorescent dye for 1 h at RT. After wash, the nuclei were counterstained with DAPI, the cells were mounted with ProLong Diamond Antifade Mountant and subjected to microscopic investigation with a confocal laser scanning microscope (ZEISS LSM780, core facility of IBMS, Academia Sinica). The antibodies and the chemicals used are listed in Table S1, S2, and Table S5, respectively.

## Tissue microarray and immunohistochemical (IHC) staining

Histopathology of all clinical samples was reviewed and confirmed by at least 2 pathologists. Representative cores (1-mm-diameter) from each tumor sample were selected by matching histology from original hematoxylin and eosin (H&E)-stained slides. IHC staining was performed using an automated immunostainer (Ventana Discovery XT autostainer, Ventana, USA) with heat-induced antigen retrieval and antibody staining procedure according to the manual instructions. Protein signal was developed using 3, 3’-diaminobenzidine (DAB) peroxidase substrate kit (Ventana, USA). The IHC staining results were scored independently by two pathologists, who were blinded to patients’ clinical outcomes. Consensus decision was made when there was an inter-observer discrepancy. For scoring, both intensity and percentage of protein expression were recorded. The staining intensity was scored as: 0, no staining; 1, weak staining; 2, moderate staining; 3, strong staining.

For survival analysis, the staining intensities were divided into “low” and “high”, where “low” includes scores 0 and 1, and “high” includes 2 and 3.

The antibodies and the chemicals used are listed in Table S1, S2, and Table S5, respectively.

## Mass spectrum analysis of proteins bound to BMI-1

The Mass protein ID analysis was performed in Proteomics Core Facility (IBMS, Academia Sinica), following the guideline provided by the facility. Briefly, after co-IP of proteins bound to BMI-1 using HA antibody, the protein samples were resolved in SDS-PAGE and stained with coomassie blue (Figure S2G). The gels were excised and sliced to thin cubes (1 mm x 1 mm), washed for 3 repeated cycles with 50 mM of triethylammoniun bicarbonate (TEABC) (15 min), and followed by 25mM TEABC containing 50% acetonitrile (15 min). The gels were then incubated in 100% acetonitrile with vigorous vortex for 15 min, and then dried in a speed vacuum. The samples were then treated with a reducing solution (20 mM dithiothreitol (DTT) in 50 mM TEABC) at 60 ℃ for 1 hr, and then treated with alkylating solution (55 mM iodoacetamide (IAA) in 50 mM TEABC) in the dark for 45 min. The samples were then washed for 3 repeated cycles with 50 mM TEABC (vortex for 15 min), followed by 100% acetonitrile (10 min), and then dried in a speed vacuum. The in-gel digestion of protein samples was performed by incubating the gels in 50 mM TEABC containing trypsin (trypsin : sample = 1 : 50 by weight) at 37℃ for 16 H. The digestion solution (containing a part of peptide samples) was transferred to a new tube. The peptide samples remaining in the gels were extracted by 3 repeated cycles with an extraction solution (5% acetonitrile and 0.1% trifluorocetic acid, vortex for 15 min) and followed by a second extraction solution (50% acetonitrile and 0.1% trifluorocetic acid, vortex for 15 min). The gel was finally extracted with 100% acetonitrile (vortex for 15 min). The digestion and extraction solutions collected in all above steps were mixed in a tube, and the peptides inside were dried in a speed vacuum, and then proceeded to LC/MS/MS Protein Identification analysis (ESI-High Resolution Tandem MS, Thermo Orbitrap Elite, IBMS, Academia Sinica).

## The syntheses of Lisuride derivatives

**General Procedure.** Reagents were used as purchased without further purification. Analytical thin-layer chromatography (TLC) was performed on precoated plates (silica gel 60 F-254), purchased from Merck Inc. Purification by gravity column chromatography was conducted using Merck Reagents Silica Gel 60 (particle size of 0.063-0.200 mm, 70-230 mesh ASTM). Proton NMR spectra were recorded on a Bruker (500 MHz) spectrometer with CDCl3 and DMSO-d6 as solvents. Multiplicities are abbreviated as follows: s, singlet; d, doublet; t, triplet; q, quartet; m, multiplet; J, coupling constant (hertz). ESI-MS spectra were recorded with an Applied Biosystems API 150EX mass spectrometer. The purities of the compounds were greater than 95% as determined by HPLC.

**Synthesis of N-(3-(1H-indol-7-yl)phenyl)-4-ethylbenzamide (Compound #44)**


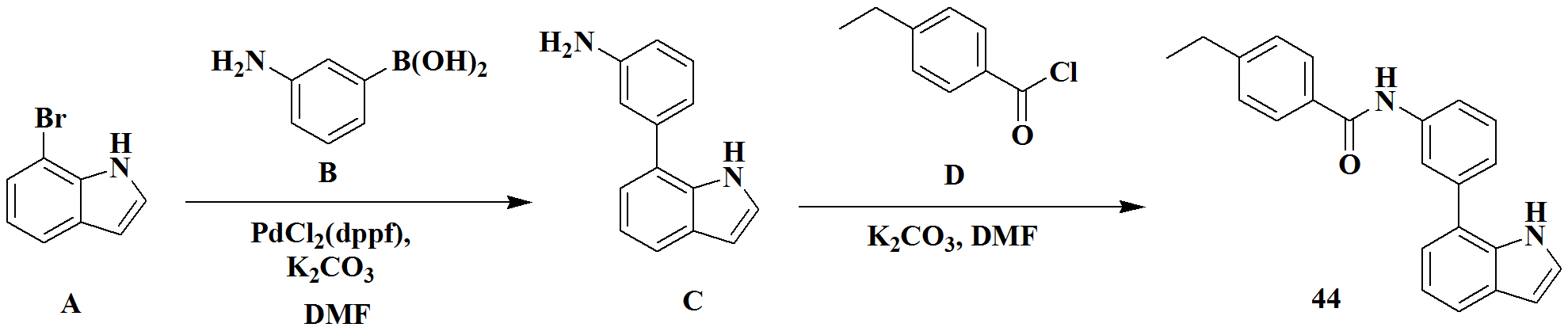


7-Bromo-1H-indole (A, 1.0 g, 5.10 mmol), 3-Aminobenzeneboronic acid monohydrate (B, 948.5 mg, 6.12 mmol) and K_2_CO_3_ (2.82 g, 20.40 mmole) in DMF (16 ml) was degassed and then flushed with nitrogen. Then PdCl_2_(dppf) (416.6 mg, 0.510 mmol) was slowly added to the solution. The reaction mixture was heated to 100°C and stirred for 5.0 hours. The reaction was monitored by TLC, and the reaction mixture was filtered with celite. The filtrate was extracted twice with ethyl acetate and the organic layer was washed with brine, dried over MgSO_4(s)_, and concentrated under reduced pressure. The residue was purified by column chromatography on silica gel to provide 3-(1H-indol-7-yl)benzenamine (**C**, 964.7 mg) in 91% yield. ESI-MS *m/z* 208.96 [M+H^+^]. ^1^H NMR (500 MHz, DMSO-*d*_6_) δ 7.37-7.36 (m, 2H), 7.15-7.10 (m, 2H), 7.01( d, *J*=7.2 Hz, 1H), 6.90 (s, 1H), 6.80 (d, *J*=7.6 Hz, 1H), 6.58-6.56 (m, 2H), 5.12 (s, 2H).

To a solution of 3-(1H-indol-7-yl)benzenamine (**C**, 137.0 mg, 0.658 mmol) in DMF (2.0 ml) was added K_2_CO_3_ (136.4 mg, 0.987 mmol) and 4-ethylbenzoyl chloride (0.145 ml, 0.987 mmol). The reaction mixture was stirred at 55 ^o^C for 4.0 hours and then quenched with water. The solution was concentrated under reduced pressure, and extracted with ethyl acetate. The organic layer was washed with brine, dried over MgSO_4(s)_, and concentrated under reduced pressure to give N-(3-(1H-indol-7-yl)phenyl)-4-ethylbenzamide (**Compound #44**, 122.2 mg) as yellow solids in 55% yield. ESI-MS *m/z* 341.60 [M+H^+^]. ^1^H NMR (500 MHz, DMSO-*d*_6_) δ 10.95 (s, 1H), 10.26 (s, 1H), 8.06 (s, 1H), 7.93-7.92 (d, *J*=7.6 Hz, 3H), 7.57-7.56 (d, *J*=7.0 Hz, 1H), 7.51-7.48 (t, *J*=7.7 Hz, 1H), 7.39-7.36 (m, 3H), 7.3 (s, 1H), 7.13-7.11 (m, 2H), 6.54-6.53 (m, 1H), 2.72-2.67 (m, 2H), 1.23-1.19 (m, 3H).

## Lung cancer xenograft in mouse model

For subcutaneous xenograft, LAC cells transduced with the scramble (SC) or shBMI1 vectors were subcutaneously injected into the flank region of mice (1 × 10^6^ cells/mouse). Tumor sizes were measured weekly with a dial caliper. Mice were sacrificed 5 weeks after injection, and tumors were resected, weighed, and photographed.

For orthotopic xenograft, H1975-Luc was constructed by stably transducing the luciferase expression pGL3-Promoter in H1975 cells. The mice were implanted orthotopically with H1975-luc by direct injection of cells to the left thorax (5 × 10^6^ cells/100 μl/mouse) from dorsal side. The tumor development was surveyed by non-invasive bioluminescent imaging after IP injection of D-luciferin (150 mg/kg), using Xtreme^TM^ Imaging System (BUX00076, Bruker, located in National Taiwan University Animal Resource Center) according to PerkinElmer technical note.

The tumor inhibition rate was calculated using the formulas as followed:

For Figure 6E: Inhibition rate = 100% x (1 – (I_e_/I_c_))

For Figure 6I: Inhibition rate =100% x (1 – (G_e_/G_c_))

where:

I_e_ indicates the Bioluminescent intensity of experiment group

I_c_ indicates the Bioluminescent intensity of control group

G_e_ indicates the relative growth rate of experiment group

G_c_ indicates the relative growth rate of control group

| Table S1, List of primary antibodies | | | |
| --- | --- | --- | --- |
| Antigen | **Manufactory/Cat. Number** | **Host** | **Application** |
| β-ACTIN | Genetex/GTX100313 | Rabbit | WB (1:2000) |
| AKT | Abcam/ab32038 | Rabbit | WB (1:3000) |
| BMI-1 | Millipore/05-637 | Mouse | WB (1:1000) |
| BMI-1 | Proteintech/ 10832-1-AP | Rabbit | IF (1:200), IHC (1:100) |
| Caspase 3 | Cell Signaling/#9664 | Rabbit | WB (1:1000) |
| EGFR | Epitomics/P11068 | Rabbit | WB (1:1000) |
| Flag epitope tag | Sigma/F3165 | Mouse | WB (1:1000) |
| GAPDH | Genetex/GTX100118 | Rabbit | WB (1:5000) |
| HA epitope tag | BioLegend/901503 | Mouse | IP (1 μg antibody/500 μg protein), WB (1:1000) |
| HISTONE3 | Abcam/ab1791 | Rabbit | WB (1:750) |
| HUWE-1 | Bethyl/A300-486A | Rabbit | IP (1 μg antibody/500 μg protein), WB (1:1000) |
| JNK1 | Cell Signaling/#3708 | Mouse | WB (1:1000) |
| JNK2 | Cell Signaling/#4672 | Rabbit | WB (1:1000) |
| Lamin A/C | Sainta-Cruz/sc-7292 | Mouse | WB (1:400) |
| MCL1 | Abcam/ab32087 | Rabbit | WB (1:1000), IF (1:200), IHC (1:100) |
| p16INK4A | Santa Cruz/SC-468 | Rabbit | WB (1:500) |
| pAKT | Abcam/ab81283 | Rabbit | WB (1:6000) |
| pEGFR(Y1068) | Cell Signaling/#3777 | Rabbit | WB (1:1000) |
| pJNK | Cell Signaling/#4668 | Rabbit | WB (1:1000) |
| PTEN | Cell signaling/#9188 | Mouse | WB (1:1000) |
| SLUG | Cell signaling/#9585 | Rabbit | WB (1:1000) |
| SNAIL | Abcam/ ab180714 | Rabbit | WB (1:1000) |
| TWIST | Cell Signaling/#46702 | Rabbit | WB (1:1000) |
| α-Tubulin | SIGMA/T9026 | mouse | WB (1:2000) |

IF, immunofluorescence. IHC, immunohistochemistry. IP, immunoprecipitation. WB, western-blot.

| Table S2, List of Secondary antibodies | | | | |
| --- | --- | --- | --- | --- |
| Antigen | **Manufactory** | **Host** | **Conjugation** | **Application** |
| Mouse IgG | Millipore/12-349 | Goat | HRP | WB (1:50000) |
| Rabbit IgG | Millipore/12-348 | Goat | HRP | WB (1:50000) |
| Rabbit IgG | Rockland/611-742-127 | Donkey | Dylight 549 | IF (1:500) |

| Table S3, List of shRNA sequence | |
| --- | --- |
| Target gene | Sequence |
| AKT1 | GGACTACCTGCACTCGGAGAA |
| AKT2 | TACCGCCCAGTCCATCACAAT |
| AKT3 | CCAAAGCCAAACACATTTATA |
| BMI1 | CAGATTGGATCGGAAAGTAAA |
| ERK1 | CCTGAATTGTATCATCAACAT |
| ERK2 | CAAAGTTCGAGTAGCTATCAA |
| HUWE1 | CGACGAGAACTAGCACAGAAT |
| JAK1 | GAGACTTCCATGTTACTGATT |
| JAK2 | GCAGAATTAGCAAACCTTATA |
| JAK3 | CTCTTCACCTACTGCGACAAA |
| JNK1 | GACTCAGAACACAACAAACTT |
| JNK2 | CTAACTTATGTCAGGTTATTC |
| MCL1 #1 | GCAGAAAGTATCACAGACGTT |
| MCL1 #2 | GAAATTCTTTCACTTCATT |
| Scramble | CCTAAGGTTAAGTCGCCCTCG |
| TRIM21 | TGGCATGGTCTCCTTCTACAA |
| UBR4 | GCCGACTAGATAGAACTGAAA |

| Table S4, List of qPCR primers | | |
| --- | --- | --- |
| Target gene | **Sequence** | **Detection** |
| 18s | F: GCAATTATTCCCCATGAACG  R: GGGACTTAATCAACGCAAGC | SybrGreen |
| 18s | F: TGG CTC ATT AAA TCA GTT ATG  R: CGG CAT GTA TTA GCT CTA | CGCTCGCTCCTCTCCTACTTG (Customized Probe) |
| AKT1 | F: GGCCCAACACCTTCATCAT  R: GATGGCGGTTGTCCACTC | UPL Probe #27 |
| AKT2 | F: TGGATTCTCCAGACGAGAGG  R: ACTTGTAGTCCATGGGGTCCT | UPL Probe #57 |
| AKT3 | F: TTGCTTTCAGGGCTCTTGAT  R: CATAATTTCTTTTGCATCATCTGG | UPL Probe #22 |
| BMI1 | F: TTCTTTGACCAGAACAGATTGG  R: GCATCACAGTCATTGCTGCT | UPL Probe #63 |
| ERK1 | F: CCCTAGCCCAGACAGACATC  R: GCACAGTGTCCATTTTCTAACAGT | UPL Probe #16 |
| ERK2 | F: AACCCACACAAGAGGATTGAA  R: TGTCGAACTTGAATGGTGCT | UPL Probe #50 |
| HUWE1 | F: GAAACCATCCCTGCCCTACC  R: GCTGCAGTTTGTTCCCCTTG | SybrGreen |
| JAK1 | F: TGCTCCTGAGTGTGTTGAGG  R: ATTTCCCAGAGCGTGGTTC | UPL Probe #5 |
| JAK2 | F: GGTGAAAGTCCCATATTCTGGT  R: AGGCCACAGAAAACTTGCTC | UPL Probe #50 |
| JAK3 | F: CTACGCCCTCAACTATCTGGA  R: TTCCGGGCAGAGACATTG | UPL Probe #43 |
| JNK1 | F: GGGCAGCCCTCTCCTTTA  R: CATTGACAGACGACGATGATG | UPL Probe #89 |
| JNK2 | F: GATATTCCAAGGCACTGACCA  R: TTCCTCACAGTTGGCTGAAGT | UPL Probe #33 |
| MCL1 | F: AAGCCAATGGGCAGGTCT  R: TGTCCAGTTTCCGAAGCAT | UPL Probe #4 |
| p16INK4A | F: CGACCGTAACTATTCGGTG  R: CCTCCTCTACCCGACCC | SybrGreen |
| TRIM21 | F: ATTCACGCAGAGTTTGTGCAG  R: GTGGACTGCACATGTCCTCA | SybrGreen |
| UBR4 | F: CCCCTGAGTGAGGACAAGG  R: AAGGAAAGGTACGGGATGATG | UPL Probe #1 |

F, Forward primer. R, Reverse primer. UPL, Universal ProbeLibrary (Roche Molecular Systems)

| Table S5, Chemicals, Biomaterials, and Aparatus | | |
| --- | --- | --- |
| Antigen | **Manufactory** | **Identifier** |
| Afatinib | Santa Cruz | sc-364398 |
| AG490 | Santa Cruz | sc-202046 |
| AlarmaBlue | BIO-RAD | BUF012A |
| chamber slides | Thermo Fisher Scientific | 154534 |
| Cisplatin | Santa Cruz | sc-200896 |
| DC Protein Assay Reagent | BIO-RAD | #5000114 |
| DMEM medium | Thermo Fisher Scientific | 11965-175 |
| DMEM/F12 medium | Thermo Fisher Scientific | 11320-082 |
| Doxorubicin | Santa Cruz | sc-280681 |
| Dynabeads (Protein A kit) | Thermo Fisher Scientific | 10006D |
| EGF | R&D | 236-EG |
| F12 medium | Thermo Fisher Scientific | 11765-047 |
| FBS | Thermo Fisher Scientific | 10437-028 |
| FGF | R&D | 233-FB |
| FluoroBlok (Trans-well insert) | Corning | 351152 |
| Gefitinib | Santa Cruz | sc-202166 |
| GENEzol | Geneaid | GZR200 |
| Hoechst | Invitrogen | H21486 |
| hydrocortisone | Sigma-Aldrich | H4001 |
| ImageQuant LAS 4000 | GE | 28955810 |
| Immobilon Western Chemiluminescent HRP Substrate | Sigma-Aldrich | WBKLS0050 |
| ITS | Thermo Fisher Scientific | 41400045 |
| jetPEI | Polyplus-transfection | 101-01N |
| Lisuride | TOCRIS | 4052 |
| LY294002 | Santa Cruz | sc-391584 |
| Matrigel | Corning | 354234 |
| MG132 | Sigma-Aldrich | M8699 |
| N2 Supplement | Thermo Fisher Scientific | 17502001 |
| Non-essential amino acids | Thermo Fisher Scientific | 11140035 |
| penicillin/streptomycin | Thermo Fisher Scientific | 10378016 |
| PhosSTOP | Sigma-Aldrich | 4906837001 |
| ProLong Diamond Antifade Mountant | Thermo Fisher Scientific | P36970 |
| PVDF | Millipore | ISEQ00010 |
| RPMI | Thermo Fisher Scientific | 11875093 |
| SP600125 | Santa Cruz | sc-200635 |
| Superscript III reverse transcriptase | Invitrogen | 18080085 |
| SuperSignal_West Pico Chemiluminescent Substrate | Thermo Fisher Scientific | 34578 |
| SybrGreen MasterMix | Thermo Fisher Scientific | A25918 |
| U0126 | Santa Cruz | sc-222395 |
| Water Sonication | Bioruptor^TM^ | UCD-200 |

# Supplementary Data

## Figure S1. BMI1 was positively regulated by JNK pathway in LAC cells


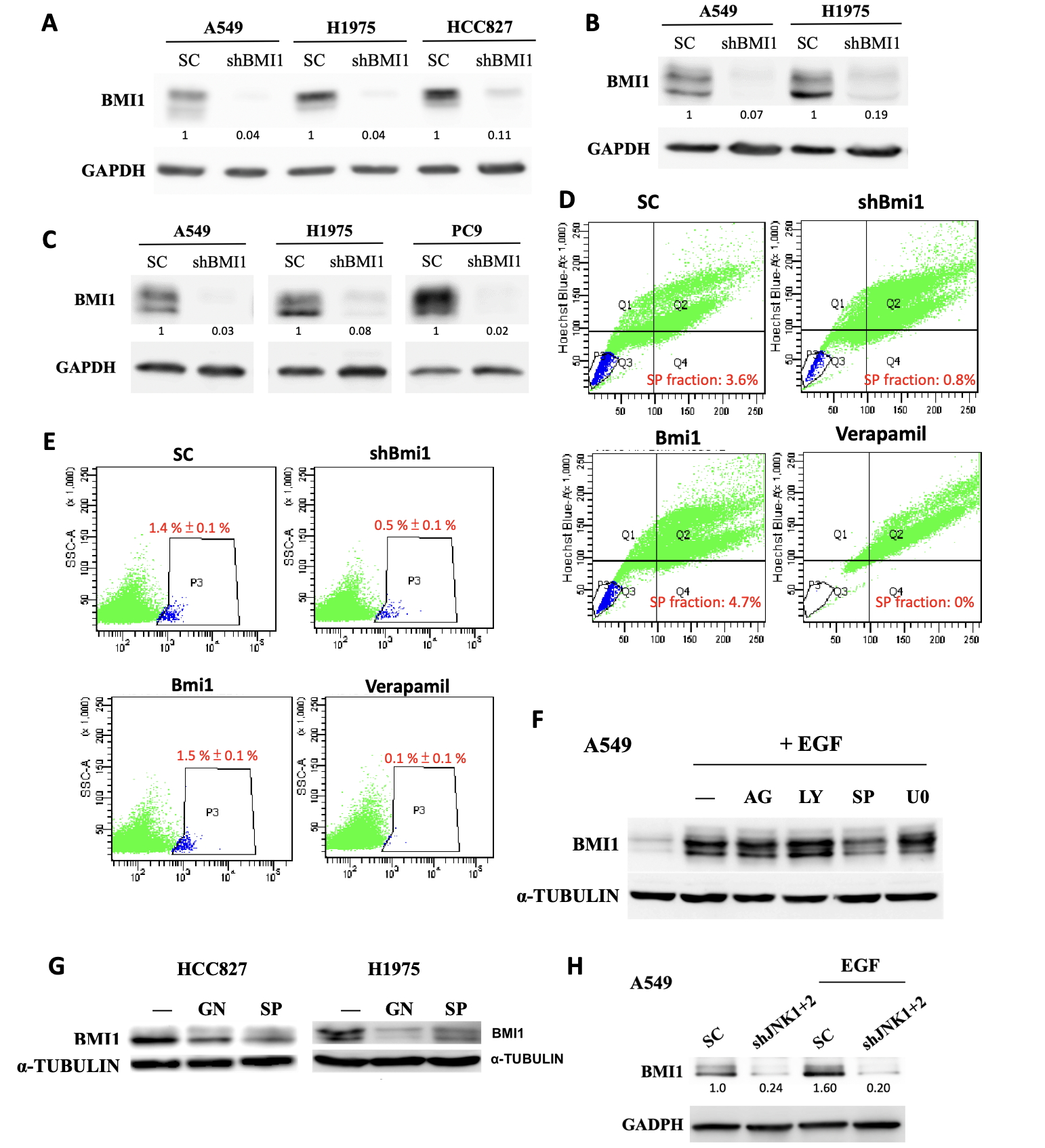


The knockdown efficiencies of BMI1 in LAC cell lines used in the experiments of Figure 1A-C (A), Figure 1D (B), and Figure 1E (C) were verified by western-blot. Knockdown of BMI1 decreased cancer stemness as shown by (D) reduced side population of cells that efflux the DNA binding dye Hoechst 33342, and (E) reduced ALDH activity in A549 cells. (F) A549 cells were pre-treated with different kinase inhibitors (10 μM, 6 h), and then treated with EGF for an additional 24 h. The results showed that only SP (JNK inhibitor) inhibited EGF-induced BMI1 upregulation. (G) HCC827 and H1975 (LAC cell lines containing constitutively active EGFR mutations) cells were treated with GN (10 nM for HCC827 and 10 μM for H1975, 24 h) or SP (10 μM, 24 h), and detected for BMI1 expression by western-blot. (H) A549 cells were transduced with shJNK1+2, with or without EGF treatment (100 ng/ml, 24 h), and then analyzed for BMI1 expression by western-blot. The results showed that shJNK1+2 blocked the EGF-mediated BMI1 upregulation.

The knockdown efficiency was detected 72 h after infection of shRNA viral vectors.

ALDH: aldehyde dehydrogenase. AG: AG490, a JAK/STAT3 inhibitor. LY: LY294002, a PI3K/AKT inhibitor. SP: SP600125, a JNK inhibitor. U0: U0126, an ERK1/2 inhibitor. GN: Gefitinib, an EGFR TKI. SC, the scramble shRNA; shJNK1+2, the mixture of shRNAs targeting JNK1 and JNK2

## Figure S2. BMI-1 was regulated mainly in post-transcriptional level in LAC cells


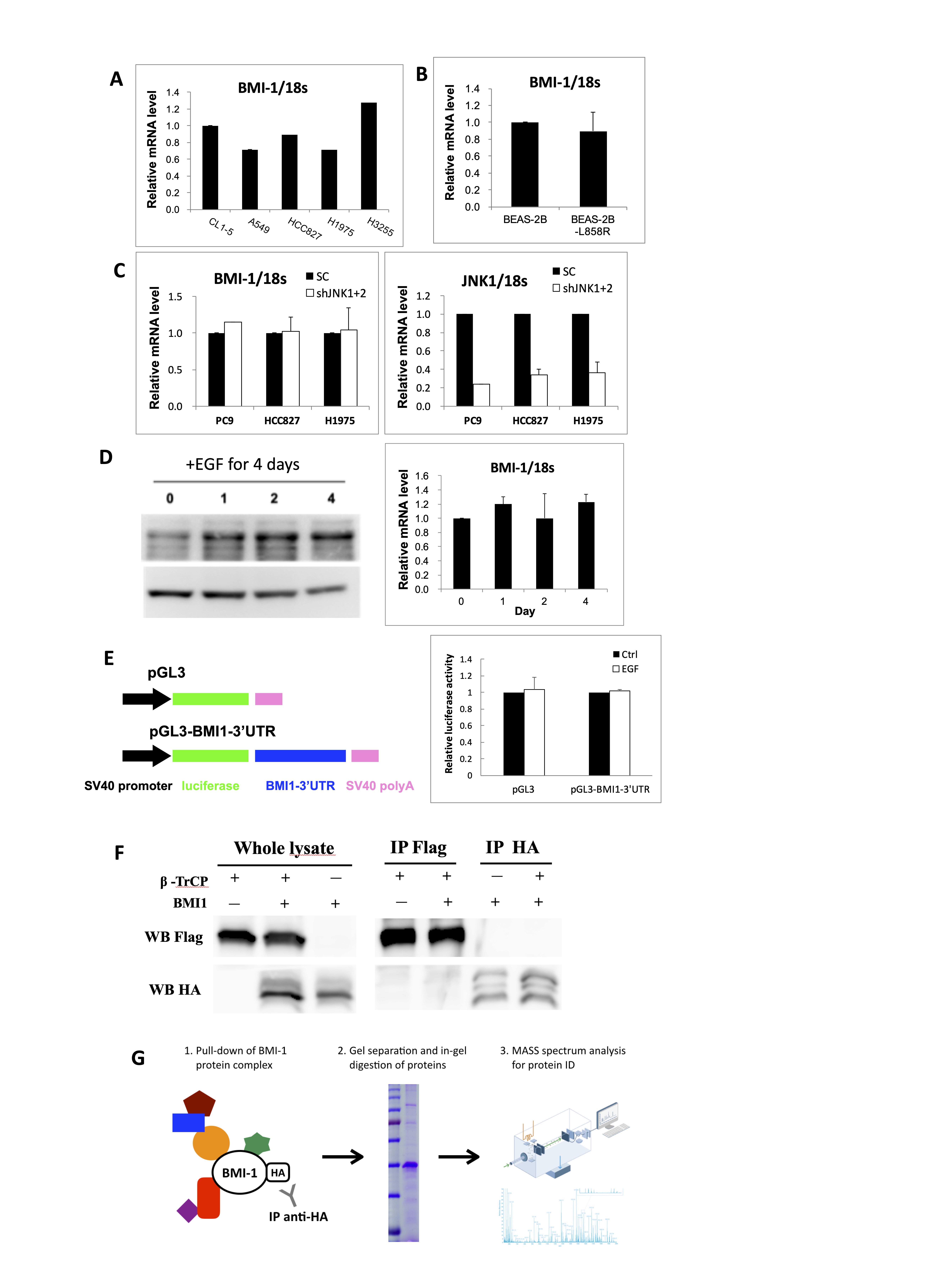


(A) BMI1 mRNA levels in LAC cell lines were detected by Q-PCR. (B) BMI1 mRNA level was detected in BEAS-2B cells with or without overexpression of mutant EGFR (L858R). (C) BMI1 (left) and JNK1 (right) mRNA levels in LAC cell lines were detected by Q-PCR after transduced with SC or shJNK vectors. (D) BMI1 protein (left) and mRNA (right) levels were detected in A549 cells after treated with EGF (100 ng/ml) for 4 continuous days. (E) BMI1 3’-UTR was constructed downstream to a luciferase reporter gene (pGL3-BMI1-3’UTR). A549 cells were detected for luciferase activity after transduced by pGL3 or pGL3-BMI1-3’UTR, with or without EGF treatment (100 ng/ml, 24 h). (F) Flag-β-TrCP and HA-BMI1 were expressed in A549 cells. Immunoprecipitation showed no direct physical interaction between β-TrCP and BMI1. (G) To analyze the proteins complexed with BMI1, A549 cells were transduced with BMI1-HA vector, and the BMI1 protein complex was precipitated by HA antibody, separated in SDS-PAGE gel, dissected and digested, and subjected to MASS spectrum for protein ID identification.

## Figure S3. The post-transcriptional interactions between JNK, HUWE1, BMI1, and MCL1 in LAC cells


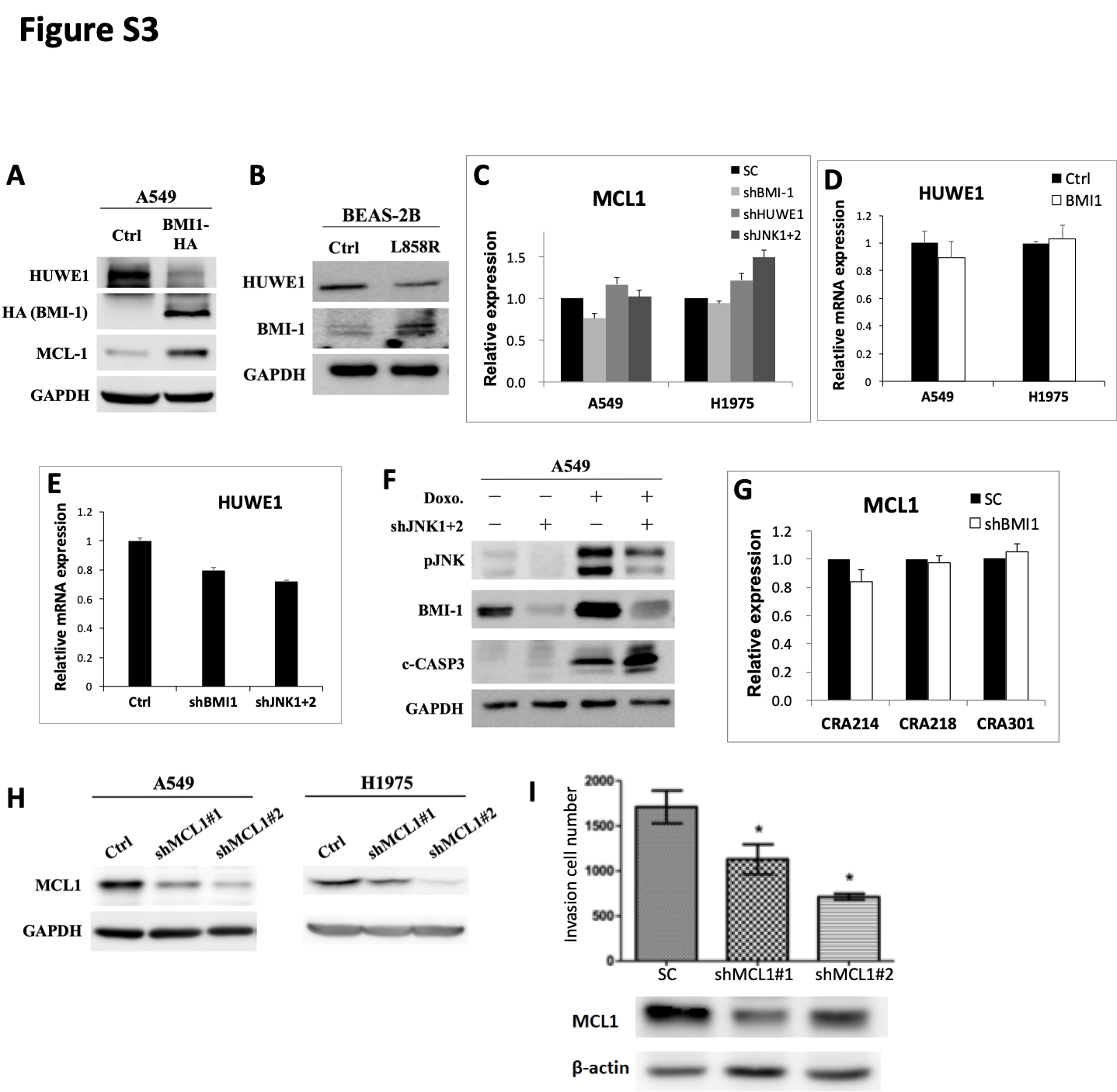


(A) HUWE1 and MCL1 expressions were detected by western-blot after overexpression of BMI1-HA in A549 cells. (B) HUWE1 and BMI1 expressions were detected by western-blot after overexpression of mutant EGFR (L858R) in BEAS-2B cells. (C) MCL1 mRNA expression was detected by Q-PCR in LAC cells after transduced with SC, shBMI1, shHUWE1, or shJNK1+2 vectors. (D) HWUE1 mRNA expression was detected by Q-PCR in LAC cells after transduced with Ctrl or BMI1 overexpression vectors. (E) HUWE1 mRNA expression was detected by Q-PCR in H1975 cells after transduced with SC, shBMI1, or shJNK1+2 vectors. (F) A549 cells were transduced with SC or shJNK1+2, with or without Doxorubicin (10 μM, 24 h) treatment, and then analyzed for pJNK, BMI1, and c-CASP3 expressions by western-blot. (G) MCL1 mRNA expression was detected by Q-PCR in CRA cells after transduced with SC or shBMI1 vectors. (H) The knockdown efficiencies of MCL1 in LAC cell lines used in the experiments of Figure 4F were verified by western-blot. (I) Cell invasion ability of A549 cells transduced with SC or shMCL1 shRNAs. The knockdown efficiencies of shMCL1 were verified by western-blot.

Doxo., Doxorubicin. CRA, Cisplatin-resistant A549

## Figure S4. Knockdown of BMI1 did not modulate the expressions of PTEN, pAKT, or p16INK4A in LAC cells

(A) PTEN and pAKT expressions were detected by western-blot in LAC cells after transduced with SC or shBMI1 vectors. (B) p16INK4A protein and (C) mRNA expressions were detected in LAC cells after transduced with SC or shBMI1 vectors. (D) TWIST, SLUG, and SNAIL expressions were detected by western-blot in LAC cells after transduced with SC or shBMI1 vectors. (E) The knockdown efficiencies of BMI1 in these experiments were confirmed by Q-PCR.

## Figure S5. The confocal microscopy of BMI1 and MCL1 expressions in LAC cells

Intracellular expressions of BMI1 and MCL1 in A549 cells were investigated by confocal microscopy after (A) knockdown or (B) overexpression of BMI1. Nuclei were counter-stained by DAPI.

SC, the scramble shRNA; shBMI1, the shRNA targeting BMI1; Ctrl, the control vector that expressed RFP; BMI1, the overexpression vector of BMI1

## Figure S6. The development of therapeutic agents targeting BMI1 and MCL1 for LAC treatment

(A) The structure of Lisuride. (B) BMI1 was detected by western-blot in H1975 after treated with different concentrations of Lisuride. (C) H1975 cells were analyzed for spheroid forming activity in serum-free matrigel, after treated with different concentrations of Lisuride. (D) The general structure of modified Lisuride as lead for optimization. The fused ring of the main core was opened to reduce its planarity, and several high polarity groups were introduced to reduce its lipophilicity. X can be N or C, Y can be CH_2_, NH or O. R^1^ and R^2^ can be high water-soluble moieties. (E) The anti-BMI1/MCL1 efficacies of Lisuride derivatives were tested in vitro by western-blot after treated in H1975 cells (10 μM, 6 h). More than 100 derivatives of Lisuride were synthesized and tested, and only a part of results was illustrated. (F) The anti-BMI1/MCL1 efficacy of the derivative #44 was tested by western-blot after treated in H1975 cells with different concentrations for 6 h. (G) Mice were orthotopically implanted with H1975-luc cells (5 × 10^6^ cells/mouse), and started to receive drug treatments for 3 weeks. The tumor growths were followed by non-invasive bioluminescent imaging, and quantified. Lisuride and Compounds #43 – 45 were administrated by IV injection through tail vein (1 mpk, 5 times/7 days). Tarceva (Gefitinib) was administrated orally (20 mpk, 5 times/7 days). N=5 to 7 for each group. The mice body weights were recording during the experiments described in Figure 6A (H) and Figure 6F (I), respectively. (J) The phosphorylation state of AKT, ERK, JAK, and JNK kinases were detected and quantified after treatments of different concentrations of BI-44. The quantification was averaged from 3 independent experiments.

mpk, mg per kg of body weight.

## Figure S7. The Schematic diagram of regulation networks between JNK, BMI1, and MCL1 in LAC

The cancer stemness could be induced by oncogenic pathway (EGFR) or environmental stress (chemotherapy) in NSCLC. Targeting BMI1 with novel small molecules could block cancer stemness and inhibit tumor progression.

# Appendix

## Molecular markers used in Western-Blot

To define the correct band of target protein in Western-Blot analysis, membranes were imaged for chemoluminescent signal (right, for target protein) as well as normal light signal (left, for markers) without moving. Both images were then put on a PowerPoint slide side-by-side to recognize the correct band according to the position relative to molecular weight markers. Main proteins investigated in this study are illustrated below.
